# Effect of Different Signal Peptides on the Expression of Glucoamylase from *Aspergillus awamori* in the Filamentous Fungus *Penicillium verruculosum*

**DOI:** 10.3390/jof12020085

**Published:** 2026-01-27

**Authors:** Nikita Eroshenko, Andrey Chulkin, Pavel Volkov, Ivan Zorov, Anna Dotsenko, Igor Shashkov, Arkady Sinitsyn, Aleksandra Rozhkova

**Affiliations:** 1 Federal Research Centre “Fundamentals of Biotechnology” of the Russian Academy of Sciences, Moscow 119071, Russia; 2 Department of Chemistry, M.V. Lomonosov Moscow State University, Moscow 119991, Russia

**Keywords:** glucoamylase, signal peptide, protein secretion, *Penicillium verruculosum*

## Abstract

Filamentous fungi are widely used in biotechnological processes because they secrete significant amounts of protein, use inexpensive nutrient media, and are predictably scalable in technological processes. *Penicillium verruculosum* B1-537 (now renamed *Talaromyces verruculosus*) produces large amounts of secreted protein (up to 70 g/L) and is used for large-scale enzyme production. Although *P. verruculosum* has an excellent protein expression system under the control of a strong *cbh1* promoter, some heterologous enzymes such as *Aspergillus awamori* glucoamylase (aaGlaA) are still produced in insufficient quantities (15–20% of the total secreted protein), and this limits the application of enzyme preparations derived from *P. verruculosum* strains in the alcohol industry for the enzymatic treatment of grain starch together with α-amylase. One of the well-known approaches to addressing this is signal peptide replacement to increase protein expression. Therefore, the aim of this study was to investigate the effectiveness of signal peptide replacement. Various signal peptides (SPs), which were previously used for other well-expressed heterologous proteins, such as xylanases, β-glucosidases, and others, were analyzed to determine their effect on aaGlaA secretion. Five plasmids containing signal peptide sequences fused to the *glaA* gene were constructed and used to transform *P. verruculosum*. The resulting strains were cultured and screened for protein content and glucoamylase activity. Copy number analysis was performed on the most productive strains. The best one was an SP of homologous glucoamylase in *P. verruculosum* (pvGlaA). The use of this particular SP increased the secretion of heterologous aaGlaA by 2.5 times when cultivating recombinant strains on cellulose-containing fermentation media for *P. verruculosum*. Thus, SP replacement is a useful way to increase expression levels in the *P. verruculosum* expression system. Application of this method in *P. verruculosum* could address some productivity issues and enable the large-scale production of other industrial and food enzymes.

## 1. Introduction

The use of filamentous fungi in industrial biotechnology is a well-established global practice due to a number of advantages of fungal expression systems, including the high secretory capacity of their microorganisms, low cost of nutrient media, and ease of isolation and purification of the final products. The economic efficiency of the microbiological production of technical, food, and feed enzymes is associated with the high productivity of recombinant fungal strains, which is a consequence of the use of DNA technologies for the heterologous secretion of in-demand enzymes with the necessary properties for specific technological processes [1,2,3].

The filamentous fungus *Penicillium verruculosum* B1-537 (VKM F-3972D, recently reclassified as *Talaromyces verruculosus*) is a highly productive cellulolytic strain producing up to 70 g/L of total secreted protein under optimized cultivation conditions. The high secretory capacity and previously developed expression system based on the strong inducible *cbh1* promoter of *P. verruculosum* allow this fungus to be used as a platform for the expression of heterologous genes and the large-scale production of enzyme preparations for technical and food purposes [4]. This system allows for the production of up to 70–80% of both homologous and heterologous target proteins (i.e., 40–70 g/L) in the total pool of secreted enzymes [5]. For example, the strain producing heterologous xylanase A (XylA) from *Penicillium canescens* was previously developed based on *P. verruculosum*. The productivity of the recombinant strain is over 60 g/L of total secreted protein, of which XylA accounts for 42–51%, and xylanase activity exceeds 3500 U/mL of cultural medium [6]. These values are 4.1 times higher than the specific activity of the native xylanase of *P. verruculosum* (F-3972). Another strain producing heterologous β-glucosidase (BG) from *Aspergillus niger* was also obtained, and the proportion of heterologous BG in the total secreted protein pool exceeds 80% [7]. In the case of *xylA* and *bgl1* gene expression, both strains used the inducible cbh1 promoter, but the signal peptides (SPs) for the production of secreted XylA and BG were their own. This indicates that the use of the fungus *P. verruculosum* (F-3972) as an expression system for enzyme production is effective and promising, and it is widely used in industrial biotechnology.

However, in some cases, the level of heterologous secretion in recombinant strains does not exceed 20%, which does not allow them to be used in the large-scale production of enzymes/enzyme preparations. Such enzymes include, for example, glucoamylase from *Aspergillus awamori* VKPM F-1262 (aaGlaA). According to our data, the secretion level of aaGlaA in *P. verruculosum* makes up only 15–20% of the total protein, and glucoamylase activity is 650 U/mL after fed-batch cultivation in a 1 L fermenter.

There are several approaches to improving the expression of heterologous genes and, consequently, increasing the productivity of target proteins in lower eukaryotes. One of them is replacing native SPs with more efficient ones (heterologous or synthetic) [3]. SPs provide protein transport to the endoplasmic reticulum (ER), as well as the secretion of target proteins into the extracellular space, which significantly facilitates the process of their isolation. Replacement of the native SP with a more effective one leads to an increase in the secretion of heterologous proteins. For example, the replacement of native α-galactosidase SP by the SP of glucoamylase in *A. niger* leads to a more-than 22-fold increase in α-galactosidase extracellular activity [8]. Similarly, the thermostable trehalase gene from *Myceliophtora thermophila* was successfully expressed in *A. niger* using this approach, to give a high yield of the target protein [9].

In addition to the major cellobiohydrolase I (CBHI), the *P. verruculosum* native secretome contains endogenous glucoamylase, pvGlaA, expressed under starch-induced conditions, suggesting that *P. verruculosum* B1-537 may be a potential glucoamylase producer for industrial applications [10]. The presence of pvGlaA in the *P. verruculosum* native secretome indicates the adaptability and high efficiency of the endogenous machinery for the processing and vesicular transport of the SPs of this enzyme. Therefore, the use of this SP is considered promising in experiments to enhance the secretion of heterologous glucoamylase from *A. awamori*. Successful precedents of a similar approach for increasing protein secretion have been described [11]. Replacing the SP for heterologous phytase from *A. niger* increased the phytase content in the preparation by 35% [12], while for heterologous mannanase from *H. jecorina*, replacing the SP increased the mannanase content in the preparation by 60% [13]. Thus, replacing the native SP of an enzyme with the peptide of a highly expressing homologous protein can lead to a significant increase in enzyme secretion levels.

Besides SPs and secretion efficiency, biosynthesis rate and intracellular stability were reported to affect the production of heterologous enzymes. For aaGlaA, production in *Saccharomyces cerevisiae* was increased by 40% through amino acid substitution of N182Q [14]. The residue N182 is the recognition site in the amino acid sequence for *N*-linked glycosylation, but it is not glycosylated in the spatial structure of aaGlaA. In the spatial structure, the residue N182 is located close to the active center of the enzyme and requires a change in conformation to form a covalent bond with an *N*-linked glycan. Therefore, in the case of glycosylation, an *N*-linked glycan can block the active center from binding with oligo- and polysaccharide substrates and cause a change in the conformation of N182. The substitution of N182Q removed the recognition site and prevented possible *N*-linked glycosylation and conformation change. The substitution did not affect the specific activity, thermostability, or proteinase resistance of the purified enzyme. Furthermore, both aaGlaA and the enzyme with the substitution of N182Q (aaGlaA*) demonstrated similar secretion efficiencies. The greater production of aaGlaA* was supposed to be due to the improvement in biosynthesis rate and intracellular stability of the enzyme [14]. The effect of the substitution of N182Q on aaGlaA* production was investigated in detail for the *S. cerevisiae* host strain. However, the preservation of this effect in other fungal host strains can be proposed due to the uniformity of the recognition site for *N*-linked glycosylation in yeast and fungal host strains, and also because the *N*-glycosylation pattern of aaGlaA produced in *S. cerevisiae, A. niger* native GlaA, and *A. awamori* var. *X100* native GlaA is reported to be the same [14].

Thus, the aim of the present study was to analyze the secretion level of *A. awamori* GlaA* influenced by various heterologous and homologous SPs in the fungus *P. verruculosum*. The signal peptides of *P. canescens* xylanase A (XylA SP) and *A. niger* β-glucosidase (Bgl1 SP) were used as heterologous SPs, while the signal sequences of *P. verruculosum* cellobiohydrolase I (CbhI SP) and glucoamylase A (pvGlaA SP) were used as homologous SPs. The efficiency of signal peptides was assessed in comparison with the GlaA secretion provided by *A. awamori*’s own GlaA peptide (aaGlaA SP).

## 2. Materials and Methods

### 2.1. Strains, Media, and Buffers

#### 2.1.1. Strains

The mycelial fungus *P. verruculosum* B1-537 (VKM F-3972, All-Russian collection of microorganisms, Puschino, Russia) was used as a host strain.

The mycelial fungus *A. awamori* VKPM F-1262 (All-Russian Collection of Industrial Microorganisms, Moscow, Russia) was used as a source of the *glaA* gene.

*Escherichia coli* MachI T1R (Thermo Fisher Scientific Inc., Waltman, MA, USA) was used in the subcloning experiments.

#### 2.1.2. Media

Minimal medium (MM): 1.5 g/L KH_2_PO_4_, 0.5 g/L KCl, 0.5 g/L MgSO_4_ × 7H_2_O, 10 g/L glucose 1%, 50 mL/L trace element solution, 10 mM NH_4_Cl.

Trace element solution (TES): 50 mg/L H_3_BO_3_, 400 mg/L CuSO_4_ × 5H_2_O, 800 mg/L FeSO_4_ × 7H_2_O, 800 mg/L MnSO_4_ × 2H_2_O, 800 mg/L Na_2_MoO_4_ × 2H_2_O, 800 mg/L ZnSO_4_ × 7H_2_O.

Fermentation medium in plates and flasks (FMP): 10 g/L KH_2_PO_4_; 1 g/L K_2_HPO_4_; 5 g/L (NH_4_)_2_SO_4_; 0.3 g/L MgSO_4_ × 7H_2_O; 0.3 g/L CaCl_2_ × 2H_2_O; 10 g/L yeast extract; 40 g/L cellulose (MCC 112); and 10 g/L wheat bran.

Fermentation medium in 1,5L-fermenter (FMF): 7 g/L KH_2_PO_4_; 5 g/L (NH_4_)_2_SO_4_; 0.3 g/L CaCl_2_ × 2H_2_O; 10 g/L yeast extract; 60 g/L cellulose (MCC 112); and 10 g/L wheat bran.

Upper agar medium (top agar): agar–agar (0.7%), 1.5 g/L KH_2_PO_4_, 0.5 g/L KCl, 0.5 g/L MgSO_4_ × 7H_2_O, 1.2 M sorbitol, glucose (0.8%), 1 mL/L trace element solution, and 10 mM NaNO_3_ as a nitrogen source (selective medium) or 10 mM NH_4_Cl (non-selective medium).

Bottom agar medium (bottom agar): agar–agar (2%), 1.5 g/L KH_2_PO_4_, 0.5 g/L KCl, 0.5 g/L MgSO_4_ × 7H_2_O, 1.2 M sorbitol, glucose (0.8%), 1 mL/L trace element solution, and 10 mM NaNO_3_ as a nitrogen source (selective medium) or 10 mM NH_4_Cl (non-selective medium).

#### 2.1.3. Buffers

ST buffer: 1.2 M sorbitol, 10 mM Tris-HCl, pH 7.0.

SCT buffer: 1.2 M sorbitol, 10 mM Tris-HCl, 10 mM CaCl_2_, pH 7.5.

PCT buffer: 50% *m*/*v* PEG-4000, 10 mM Tris-HCl, 10 mM CaCl_2_, pH 7.5.

### 2.2. Cloning of aaglaA Gene

Genomic DNA isolated by DNeasy Plant Kit (Thermo Fisher Scientific Inc., Waltman, MA, USA) from the filamentous fungus *A. awamori* VKPM F-1262 was used as a template for PCR using Phusion™ High–Fidelity DNA Polymerase (New England BioLabs Inc., Ipswich, MA, USA) and the primer pair aaGlaLICF and aaGlaLICR (Table S1) for the amplification of the *aaglaA* gene. The *aaglaA* gene was cloned to the pCBHI vector [4] using an adapted LIC-method that we used previously [15,16]. The resulting PCR fragment, approximately 2200 bp in length, was inserted into the expression vector pCBHI under the *cbh1* gene promoter with a transcription terminator for the same gene, as described previously [16]. The nucleotide and translated amino acid sequence of the *aaglaA* gene are shown in Figure S1. The absence of additional mutations, deletions, or insertions in the resulting pCbhGla plasmid was confirmed by sequencing in both directions according to Sanger et al. [17].

### 2.3. Site-Directed Mutagenesis of aaglaA Gene

**Figure 1:**
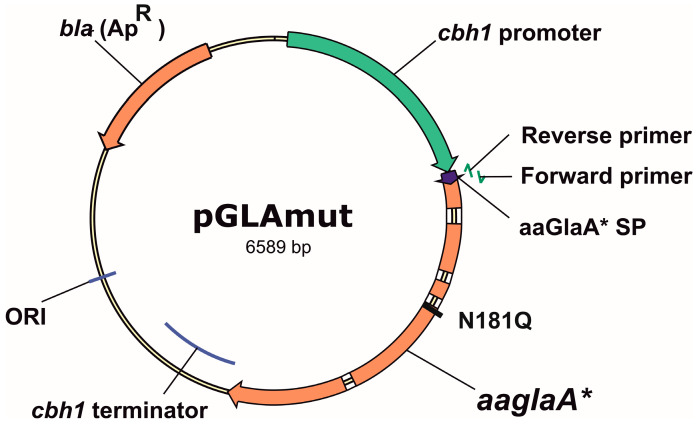
Map of pGLAmut plasmid. The plasmid shows the location of the N181Q mutation in aaglaA* gene. The variable region of the nucleotide sequence where the signal peptide substitution occurred is also indicated.

The amino acid substitution of N181Q was introduced into the amino acid sequence of aaGlaA based on the reported positive effect of an analogous substitution on glucoamylase production in *S. cerevisiae* [14]. A mutant form of aaGlaA* was generated by introducing a codon mutation into the aaglaA gene using the QuickChange method [18]. The mutation N181Q in the modified enzyme aaGlaA* were introduced into the *aaglaA* gene with N181Q-fwd and N181Q-rev primers (Table S1), resulting in a mutated aaglaA* gene. PCR was performed for the pCbhGla plasmid with the Phusion High-fidelity PCR Kit (New England BioLabs Inc., Ipswich, MA, USA). Then the parental plasmid template was cut with *Mal*I endonuclease (SibEnzyme, Novosibirsk, Russia), and the pGLAmut plasmid with the target mutations was transformed into *Escherichia coli* XL-1 Blue competent cells (Agilent Technologies Inc., Santa Clara, CA, USA) using a transformation protocol [19]. The pGLAmut plasmid with the mutated aaglaA* gene was produced in *E. coli* cells and purified (Evrogen, Moscow, Russia). The map of the resulting plasmid pGLAmut is shown in Figure 1. The sequence of the mutated aaglaA* gene was confirmed by DNA sequencing [17].

### 2.4. Creation of Plasmids with aaglaA* Linked to Different Signal Peptide Sequences

Using the pGLAmut plasmid as a template, the SP aaGlaA was replaced with the SP BGLI from *A. niger*, SP CBHI from *P. verrululosum*, SP pvGlaA from *P. verrululosum*, and SP XylA from *P. canescens* using inverse PCR.

For this purpose, pairs of primers were synthesized for each signal peptide (Table S1). The 3′-end of the forward primer was complementary to the beginning of the sequence encoding mature aaGlaA* glucoamylase, and the 3′ end of the reverse primer was inversely complementary to the 5′-end of the *cbh1* promoter. The 5′-ends of the primers overlapped and together encoded the corresponding SP.

The pGLAmut plasmid map with primers for SP replacement is shown in Figure 1.

Plasmid pGLAmut DNA was used as a template for PCR using Phusion™ High-Fidelity DNA Polymerase (New England BioLabs Inc., Ipswich, MA, USA), and a primer pair was used to replace the corresponding SP. The resulting PCR fragments, approximately 6.6 kb, were digested with the restriction endonuclease *Dpn*I (Thermo Fisher Scientific Inc., Waltman, MA, USA) and transformed into *E. coli* XL-1 Blue competent cells (Agilent Technologies Inc., Santa Clara, CA, USA).

The correct assembly of all the constructs with the replaced SP was verified by sequencing [17].

### 2.5. Transformation of the Penicillium verruculosum B1-537 Host Strain

The plasmids pCbhGla and pGlamut, as well as plasmids with substituted signal peptides, were transformed into the *P. verruculosum* B1-537 host strain in accordance with [16,20]. *P. verruculosum* B1-537 (ΔniaD) has a defect in the nitrate reductase gene (*niaD*) and is unable to metabolize nitrate nitrogen.

Briefly, the B1-537 strain was inoculated by washing spores from a Petri dish into 100 mL of MM. The cells were incubated in flasks for 12–14 h at 30 °C and 200–250 rpm. The grown mycelia were passed through a glass filter, resuspended in lysis buffer (pH 5.6–5.8) containing 15 mg/mL *T. harzianum* lysing enzyme preparation (Sigma-Aldrich, St. Louis, MO, USA) and 2.5 mg/mL bovine serum albumin, and incubated for 2–3 h at 30 °C and 200 rpm. The hydrolysate obtained was transferred to a sterile centrifuge tube, layered with ST buffer, and centrifuged, and a layer of protoplasts at the interface was picked and redissolved in SCT buffer.

Then, 3 × 10^7^ protoplasts dissolved in 200 μL SCT buffer, 10 and 1 μg of target and cotransformation DNA (plasmid pSTA10 [21] containing native *niaD* gene), respectively, and 50 μL PCT buffer were used for each transformation point. The mixture was incubated for 20 min on ice. Then, 500 µL of PCT buffer was added, incubated for 5 min at room temperature, and redissolved in 200 µL of 1.2 M sorbitol buffer.

Petri dishes with ‘bottom agar’ and test tubes with ‘top agar’ were used for seeding. The transformation mixture was added to tubes containing the top agar, which had previously been melted in a water bath. The mixture was seeded on selective medium. As a control, protoplasts without exogenous DNA were seeded on selective and non-selective media. Petri dishes containing transformants and controls were placed in a thermostat at 30 °C for 5 days.

### 2.6. Primary Screening of Recombinant Strains in Plates and Flasks

For primary screening in 24-well plates, transformants were cultured in 5 mL of FMP medium per well (30 °C, 250 rpm). Samples were collected on the 6th day. The criteria for the selection of transformants were maximum glucoamylase activity and protein concentration. SDS-PAGE was performed to confirm glucoamylase expression and to extract samples for mass spectrometric analysis.

The best transformants were screened in 750 mL Erlenmeyer flasks in 100 mL of FMP. After culturing for 144 h on a rocker at 220 rpm and 30 °C, the culture fluid (CF) was separated from the mycelium by centrifugation for 10 min at 10,000× *g*. The criteria for selection of transformants in flasks was the same as for plates. Selected recombinant clones were spread onto slants of agar supplemented with 10 mM NaNO_3_ for storage and future experiments.

### 2.7. Glucoamylase Strains Cultivation in 1.5 L-Fermenters

The most promising glucoamylase strains were cultured in 1.5 L fermenters (Prointex, Puschino, Russia) for 144 h (32 °C, 300 rpm) on FMF media. The FMF was sterilized at 121 °C (1 atm) for 1 h. The glucose solution (40 g in 100 mL of water) was sterilized by separate autoclaving at 0.5 atm for 30 min. The 1.5 L fermenter containing 700 mL of FMF was inoculated with 100 mL of inoculum under sterile conditions.

Cultivation was carried out under the following conditions: air flow of 0.4 L/(min*L), 32 °C, a lower limit of pO_2_ of 30%, an initial stirrer speed of 300 rpm (during fermentation, the speed was automatically adjusted according to the values of the pO_2_ sensor), the pH of the medium not being lower than 4.5, and retitration carried out with 10% ammonia or 5% H_2_SO_4_ solutions. After 48 h of cultivation, the fermenter was replenished with 25 mL of 50% glucose solution every 8–12 h. Microcrystalline cellulose suspension (2.1 g per 25 mL of water) was added to the fermenter at 72, 96, and 120 h of cultivation.

After 144 h, the contents of the fermenter were transferred to centrifuge containers and centrifuged at 4000 rpm for 1 h in an Avanti J-26S centrifuge (Beckman, Brea, CA, USA). The resulting supernatant was microfiltered using a Millipore TFF Pellicon 2 system and used for freeze-drying using Virtis SP Scientific 9L (SP, Warminster, PA, USA).

### 2.8. Enzymatic Activities Assay and Protein Concentration

The glucoamylase activity was determined by analyzing the glucose released after a 10 min enzyme reaction with 12 mg/mL soluble starch at pH 4.7 (0.05 M Na–acetate buffer).

The activity of the enzyme towards avicel (microcrystalline cellulose, MCC), carboxymethyl cellulose (CMC), and beechwood xylan (Sigma, St. Louis, MO, USA) was determined by measuring the accumulation rate of reducing sugars (RSs) using the Nelson–Somogyi method [22]. Enzymatic reactions were conducted for 10 min (60 min in the case of MCC) at pH 5.0 (0.05 M Na–acetate buffer) and 50 °C (40 °C in the case of MCC) with a substrate concentration of 0.5%. CMCase, xylanase, MCC, and glucoamylase activities were expressed in international units. One unit of activity corresponded to the quantity of enzyme that released 1 μmol of RS (in glucose equivalents) per minute.

The protein concentration was determined by the Lowry method [23], with bovine serum albumin (BSA) being employed as the standard.

All experiments and measurements were performed in triplicate.

### 2.9. Real-Time PCR to Determine the Number of aaglaA* Gene Copies in the Genome of Recombinant Strains and Assessment of the Transcription Level of the aaglaA* Gene

The qPCR reaction mixture consisted of 0.4 pM forward and reverse primers for the *aaglaA* and *actA* (Table S1), as well as 4 μL of a “5X qPCRmix-HS SYBR” (Evrogen, Moscow, Russia), 10 μL of the analyzed DNA/cDNA sample, and water up to 20 μL.

The qPCR pattern was as follows: first stage, 5 min at 95 °C; second stage, 39 cycles, 15 s at 95 °C and 45 s at 60 °C, with fluorescence measurement after each cycle; and third stage, an increase in temperature from 75 °C to 95 °C in 0.2 °C/10 s steps with fluorescence measurement. The melting curve of the PCR products was plotted at the third stage.

Amplification was carried out in a CFX96 amplifier (Bio-RAD, Hercules, CA, USA) in compatible 96-well plates with low-profile plates. The results were analyzed with Bio-Rad CFX Maestro v.1.1 Software (Bio-RAD, Hercules, CA, USA).

All samples were analyzed in triplicate.

To determine the copy number, 10 ng of gDNA was used. To determine the amplification efficiency, the following amounts were used: 1, 3, 10, 30, and 100 ng of gDNA of the pvGlaA SP21 strain. The *P. verruculosum* actin (*actA*) gene [24] was used as a reference gene for qPCR.

To assess the level of transcription, RNA isolated from the mycelium of strains from the fermenter at 144 h was used. Total RNA samples (1 μg) were treated with “RNAse-free DNAse I” (Thermo Fisher Scientific Inc., Waltman, MA, USA) according to the manufacturer’s recommendations. cDNA was synthesized on treated RNA (3 μL) with the “RevertAid H Minus First Strand cDNA Synthesis Kit” (Thermo Fisher Scientific Inc., Waltman, MA, USA), as well as “Random hexamer primer” and “Oligo(dT)18 primer” (Thermo Fisher Scientific Inc., Waltman, MA, USA). After the completion of the reverse transcription reaction, the samples were diluted with 150 μL of DEPT water. The resulting cDNA was used as a template for qPCR. *P. verruculosum* actin (*actA*) was used as a reference gene for qPCR in this set of experiments, as described above.

### 2.10. Component Composition of Glucoamylase Enzyme Preparations

The percentage of each enzyme in the enzyme preparation was carefully determined using FPLC analysis. Isolation and purification, including desalting by gel filtration and anion exchange chromatography, were performed using an ÄKTA Pure chromatography system (Cytiva, Marlborough, MA, USA). Gel filtration was performed on a Bio-Gel P2 column equilibrated with 20 mM Bis-Tris/HCl, pH 6.8. The desalted protein mixture was then applied to high-performance anion exchange chromatography on Mono Q beads (9 mL, Cytiva, Marlborough, MA, USA), and proteins were eluted with a slow linear gradient of 0.4 M NaCl in 20 mM Bis-Tris/HCl, pH 6.8, from 0 to 225 mM. The chromatographic peak areas of individual enzymes and their contribution to the total content of protein components were determined. The purity of the peaks was confirmed by SDS-PAGE, followed by MALDI-TOF analysis of protein bands. The specific activities of each purified enzyme were measured to confirm the correct mechanism of action.

### 2.11. Mass Spectrometric Analysis

Peptide mapping using mass spectrometric analysis was performed after trypsin (Promega, Madison, WI, USA) digestion of the protein contained in the corresponding gel band [25]. MALDI-TOF mass spectrometry of trypsin hydrolysates was performed on an UltrafleXtreme II (Bruker Daltonik GmbH, Bremen, Germany) at the Industrial Biotechnology Center of the Federal Research Center of Biotechnology, Russian Academy of Sciences. The obtained data were interpreted by comparing the masses of the obtained peptides with those theoretically calculated using the peptide mass service.

### 2.12. Statistical Analysis

All experiments and measurements were performed in triplicate. Significant differences were considered if *p*-values were below 0.05. Comparisons of values and standard deviations for activity and protein concentrations were made using Fisher’s method. (a) *p* (<0.1), (b) *p* (<0.05), (c) *p* (<0.01). Measurement errors in qPCR were calculated using Bio-Rad CFX Manager v.3.1 software (Bio-Rad Laboratories Inc., Hercules, CA, USA). Quantitative results of the enzyme activity measurements were analyzed using STATISTICA v. 6.1 software (StatSoft Inc., Tulsa, OK, USA).

## 3. Results

### 3.1. Bioinformatic Analysis of Signal Peptides and Obtaining of Plasmids with Signal Peptide Variants

From previously published data, it is known that SP substitution can positively affect the expression of extracellular proteins in filamentous fungi [8]. Using the SignalP 6.0 Program (https://services.healthtech.dtu.dk/services/SignalP-6.0/ (accessed on 13 January 2025)), we analyzed the amino acid sequence of mature aaGlaA* glucoamylase fused to the signal peptides of other proteins previously shown to be highly expressed in *P. verruculosum*, as well as fused to the signal peptide of homologous *P. verruculosum* glucoamylase, to determine the probability of SP cleavage [26]. The nucleotide and corresponding amino acid sequences of SPs are shown in Figure S2. The results of the bioinformatics analysis are presented in Table 1 and Figure S3A–E.

Bioinformatic analysis revealed that all the amino acid sequences studied are signal peptides with a probability higher than 98% (Figure S3). However, the probabilities of signal peptide cleavage during aaGlaA* secretion can be ranked as follows: pvGlaA SP > BglI SP > CbhI SP > XylA SP > aaGlaA SP (Table 1).

However, it should be noted that the use of bioinformatics analysis is based on the statistical calculation of cleavage sites across all eukaryotic microorganisms, without taking into account the biological characteristics of a particular fungus. Therefore, we performed mass spectrometric analysis of aaGlaA to confirm the correct signal peptide cleavage site (Figure S3F). Theoretical cleavage of the amino acid sequence of aaGlaA with the native SP suggests possible masses of sequences containing fragments of the SP (Table S2). Mass spectrometric analysis revealed fragments corresponding to the N- and C-termini of the amino acid chain (highlighted in yellow in Table S3). The peak with *m*/*z* 1633.819 corresponds to peptide 25–39, and the peak with *m*/*z* 2344.087 corresponds to peptide 621–640. No masses corresponding to fragments of the native aaGlaA SP were detected. These data demonstrate that, despite the relatively low calculated probability, cleavage of the native aaGlaA SP occurs precisely at the predicted point.

Thus, based on the pGlamut plasmid (Figure 1), a set of target plasmids with the gene of mutated glucoamylase, aaglaA*, and various signal peptides was obtained as described in Section 2.4, sequenced, and transferred to the transformation step.

### 3.2. Transformation of Penicillium verruculosum B1-537 Host Strain and Primary Screening of Recombinant Clones

Using the protocol described in Section 2.5, a set of transformants was obtained on selective medium supplemented by 10 mM NaNO_3_. The number of clones is given in Table 2. The transformation frequency was 5–7 clones/μg of DNA. The morphology and growth rate of the resulting clones did not change with the host strain.

To identify the most productive clone and the most effective signal peptide, a sequential screening for all clones was applied.

#### 3.2.1. Screening of Recombinant Clones in 24-Well Plates

A classical screening of all obtained clones was carried out in culture plates as described in Section 2.6. Results of the screening are presented in Table 3. Table 3 illustrates the total glucoamylase activities determined in the CF of the recombinant strains after 144 h of cultivation on the FMP media. The CF of the host strain, collected under similar conditions, was used as a control. From Table 3, it follows that not all transformants had an increased level of glucoamylase activity, which indicates the absence of integration of the target plasmids in some cases. We observed this effect [27] since the transformation of the host strain was carried out using separate plasmids, including the target aaglaA* gene and the native *niaD* gene (pSTA10).

The criteria for the selection of transformants were maximum glucoamylase activity and protein concentration. According to the results of the primary screening in plates, the most effective signal peptide was pvGlaA SP and XylA SP. However, the most active transformants towards starch from all series were selected for screening in flasks. Specifically, for the pvGlaA SP series, clones 4, 19, 21; for the XylA SP series, clones 2, 6 and 7; for the Cbh1 SP series, clone 15; for the Bgl1 SP, clones 2, 7, 12 and 25; for the aaGlaA SP series, clones 6 and 13 (all selected clones are marked in red in Table 3).

#### 3.2.2. Screening of Recombinant Clones in Flasks

**Figure 2:**
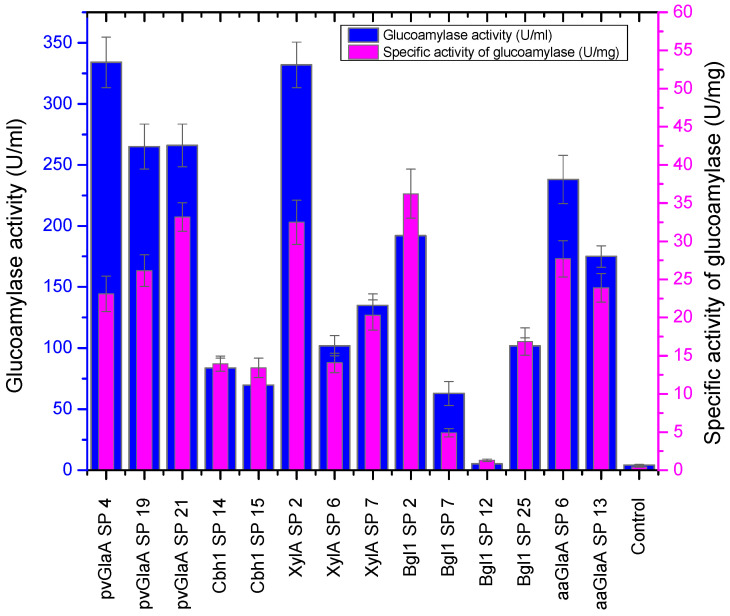
Screening of recombinant strains producing aaGlaA*. The diagram shows the total (left axis in the diagram) and specific (right axis in the diagram) glucoamylase activities toward starch for clones obtained by plasmid transformation of the *P. verruculosum* B1-537 host strain.

Selected clones were pumped into flasks with 100 mL of FMP. After 144 h, the samples of CFs were subjected to SDS-PAGE (Figure S4). Also, glucoamylase activity, total protein concentration, and the activities of basal enzymes (CMCase, Xylanase, Avicelase) were analyzed. The specific activity of glucoamylase toward starch was calculated (Table S4, Figure 2).

Based on the results of screening in flasks, the most effective signal peptides were *pvGlaA SP* and *XylA SP*. To identify the most effective clone and signal peptide, the four best strains were selected for cultivation in a 1.5 L fermenter. These were the clones pvGlaA SP21, XylA SP2, Bgl1 SP2, and aaGlaA SP6, as the clones with the highest total and specific glucoamylase activities.

### 3.3. Selected Strains Cultivation in 1.5 L-Fermenter

**Figure 3:**
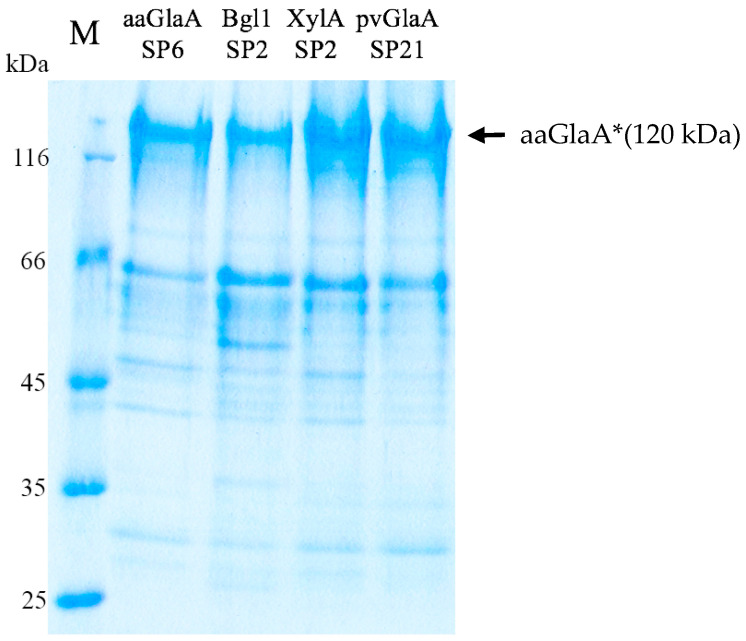
SDS-PAGE (10.5% gel) for CF samples of pvGlaA SP21, XylA SP2, Bgl1 SP2, and aaGlaA SP6 taken after 144 h of cultivation. A 10 μg protein sample was applied per track. M- protein marker. The band corresponding to the molecular weight of aaGlaA* is indicated by an arrow.

The clones were cultured in a 1.5 L fermenter as described in Section 2.7. Samples of CF were collected at 48, 72, 96, 120, and 144 h. Glucoamylase activity and protein concentration in the samples were analyzed (Table 4). SDS-PAGE was performed for the CF samples collected at 144 h of cultivation (Figure 3). Mass spectrometric analysis confirmed that the 120 kDa band corresponded to mutated aaGlaA*.

The most intense aaGlaA* band was observed in the case of pvGlaA SP21 and XylA SP2 clones based on SDS-PAGE at the same protein loading (Figure 3).

This is confirmed by data on the enzymatic activity of aaGlaA* after 144 h of cultivation. Indeed, the dynamics of aaGlaA* biosynthesis were approximately the same for all recombinant strains, and their total protein concentrations after 144 h of cultivation ranged from 27 to 30 g/L. However, by the end of the cultivation of clones pvGlaA SP21 and XylA SP2, glucoamylase activity was 2400 ± 180 and 1750 ± 120 IU/mL, respectively, which is higher than that of the other clones. (Table 4).

### 3.4. Content of Glucoamylase in Dry Enzymatic Preparations

Dry enzyme preparations were obtained according to the scheme described in Section 2.7. The preparations were separated by anion-exchange column chromatography by FPLC (Figure S5). The area of the chromatographic peaks related to GA accounted for 74–77% of the total amount of secreted protein for the two best enzyme EPs, pvGlaA and XylA. Table 5 shows the glucoamylase content in the dry EPs. The enzymatic activity of the best EP, pvGlaA SP21, is 75% higher than that of aaGlaA SP6 and 15% higher than that of EP XylA SP2.

Chromatographic analysis of enzyme preparations derived from aaGlaA* recombinant strains revealed the highest glucoamylase content in the strain using the homologous pvGlaA SP from *P. verruculosum* and the heterologous XylA SP from *P. canescens* (77.4% and 74.1%, respectively). These data correlate to the highest glucoamylase activity in these enzyme preparations (33,300 U/g and 28,600 U/g, respectively).

### 3.5. Real-Time PCR and Determining the Number of glaA Gene Copies and Transcription

The qPCR method was used to determine the number of copies of the aaglaA* gene. The actin gene (*actA*) was used as a reference gene. To perform qPCR, primers (Table S1) were synthesized and calibrated to determine their efficiency. The results were analyzed using Bio-Rad CFX Manager v.3.1 software (Bio-Rad Laboratories Inc., Hercules, CA, USA). The amplification efficiency for the aaglaA* and actA genes was 101.2% (confidence 0.998) and 99.1% (confidence 0.991), respectively. Using these values, copy number data were obtained. Figures S6 and S7 present the qPCR results describing the number of aaglaA* gene copies in the genomes of recombinant strains pvGlaA SP21 and XylA SP2.

The genome of pvGlaA SP21 contains 18–22 insertions of the gene aaglaA*, and the genome of XylA SP2 contains 29–35 insertions of the gene aaglaA*.

We also determined the transcription level of the aaglaA* gene in the aaGlaA SP6, Bgl1 SP2, pvGlaA SP21, and XylA SP2 strains. The results are shown in Figures S8 and S9. It was shown that the levels of the aaglaA* gene transcription are approximately the same in all analyzed strains.

## 4. Discussion

This study demonstrates a successful strategy for significantly enhancing the heterologous expression of *A. awamori* glucoamylase in the industrial filamentous fungus *P. verruculosum* through signal peptide engineering. Our results strongly suggest that signal peptide selection is a critical factor in the efficiency of recombinant protein secretion and can serve as a powerful tool for the metabolic engineering of fungal cell factories.

The subject of this study was glucoamylase (aaGlaA), one of the most in-demand enzymes in biotechnology. This enzyme is well known and widely used in grain processing and other microbiological industries worldwide [28]. The choice of this enzyme as a model for experiments to study the effect of signal peptides on the secretion of glucoamylase was due to the need to obtain new industrial strains based on the mycelial fungus *P. verruculosum* for use in biotechnological production in the Russian Federation.

It is also important to mention that the substitution of N181Q was performed in aaGlaA* to improve the rate of biosynthesis and intracellular stability of this enzyme, according to the reported data on the production of aaGlaA in *S. cerevisiae* [14]. In addition to the improvement in production, this substitution was reported to maintain the specific activity, thermostability, and proteinase resistance of the enzyme [14]. Similarly to the production of aaGlaA* in *S. cerevisiae*, the glucoamylase activity in the culture fluid increased continuously during the production of aaGlaA* in *P. verruculosum* B1-537 (Table 4). Therefore, the introduction of a substitution of N181Q based on that study was an appropriate approach.

Selecting an effective signal peptide as an approach is not new [29]; however, despite the wide range of industrial and food enzyme producers derived from *P. verruculosum*, the most effective signal peptide has not been identified. Prior to the studies presented in this paper, in expression plasmids for transforming the recipient *P. verruculosum* B1-537, target genes were either coupled to the *cbh1* peptide or were expressed under their own signal peptides [30,31].

According to the results of mass spectrometric analysis of aaGlaA, the amino acid chain of the secreted protein contained residues 25–640 (Table S3). In the mass spectrum (Figure S3F), a peptide corresponding to the N-terminus of the amino acid chain was detected (25-ATLDSWLSNEATVAR-39, highlighted in yellow in Table S3, numbering in the table from the first residue, M, of the signal peptide, *m*/*z* 1633.819). Also, in the mass spectrum, a peptide corresponding to the C-terminus of the amino acid chain was detected (621-EYTVPQTCGESTVTVTDTWR-640, highlighted in yellow in Table S3, numbering in the table from the first residue, M, of the signal peptide, *m*/*z* 2344.087). Peaks for peptides that could theoretically be obtained by the cleavage of the signal peptide (Table S2) were not detected in the mass spectrum. Therefore, it can be concluded that the aaGlaA SP is cleaved at a position between amino acid residues 24 and 25, and in the case of aaGlaA, we observe a canonical variant of the secretion via a signal peptide, which is most likely true for the other standard peptides used.

Thus, recombinant strains producing aaGlaA* fused to different selected SPs were obtained using a standard protocol that has previously been used in the laboratory [4]. The transformation frequency was slightly lower than usual (typically 20 transformants per 1 μg of DNA). However, a decrease in transformation frequency was observed for all plasmid constructs, which may be related to the structure of the aaglaA* gene [32]. Importantly, expression of the aaglaA* gene was controlled by the strong inducible *cbhI* promoter, which virtually eliminated the expression of the intrinsic glucoamylase, whose expression was detected only on starch-containing media [33].

Sequential screening of recombinant *P. verruculosum* strains was conducted in a manner similar to that used to obtain the producers of xylanase A and E, β-glucosidase, pectin lyase, polygalacturonase, and other enzymes, which were produced using the *P. verruculosum* expression platform [4]. A similar screening scheme is used for other expression platforms to produce industrial strains [34,35,36].

A key outcome of this work was the identification of two highly effective signal peptides (XylA SP from *P. canescens* and pvGlaA SP from *P. verruculosum*). Their use resulted in maximal levels of aaGlaA* secretion. Despite obtaining comparably high enzymatic activities (1750 U/mL for XylA SP and 1854 U/mL for pvGlaA SP, Table 4, and 28,600 U/g for XylA SP2 and 33,300 U/g for pvGlaA SP21, Table 5), the copy numbers of the integrated aaglaA* gene in their genomes differed (29–35 and 18–22 copies, respectively). Further transcriptional analysis of aaGlaA SP6, Bgl1 SP2, pvGlaA SP21, and XylA SP2 strains revealed that the transcription level of the aaglaA* gene was the same for all selected strains within the standard deviation (Figure S9). We suggest that the absolute number of aaglaA* gene copies is not as important in our case, as the transcriptional mechanism saturates due to the titration effect of positive transcription factors [37] regulating the strong inducible *cbh1* promoter. Therefore, the increase in aaGlaA* secretion levels is due to the efficiency of the signal peptides.

This observation confirms our hypothesis that expression levels are governed not only by gene copy number but also by the efficiency of the translocation and secretion processes initiated by the signal peptide. The same data were obtained in a study [38], where analysis of the influence of signal peptides glucoamylase, alpha-amylase, and cellobiohydralase on GFP expression in *P. oxalicum* led to the discovery that GFP expression under the control of glucoamylase SP was higher, and the increase in efficiency was associated only with the nature of the SP. Reference [8] also demonstrates that the level of alpha-galactosidase expression is directly related to the nature of the signal peptide and can increase severalfold when it is replaced.

Analysis of the chromatographic profiles of the enzyme preparations reveals a higher content of glucoamylase in the pool of total secreted proteins in the case of the signal peptides XylA and pvGlaA compared to the signal peptides aaGlaA and Bgl1 (Table 5), which correlates with the higher activity of glucoamylase in the corresponding enzyme preparations, pvGlaA SP 21 and XylA SP 2. The GA activity in the pvGlaA SP 21 enzyme preparation was 75% higher compared to *Aspergillus* aaGlaA SP and about 15% higher in comparison with the second-best preparation, XylA SP.

We hypothesize that the high efficiency of the pvGlaA SP and XylA SP may be attributed to their superior affinity for the signal recognition particle (SRP) and/or more efficient recognition and cleavage by signal peptidase in the endoplasmic reticulum [39,40,41,42,43]. This would facilitate faster and more efficient polypeptide translocation, thereby reducing the probability of unproductive folding or proteolytic degradation in the cytosol [44,45,46,47].

Despite the clear results, this study has certain limitations. First, the study was limited to a predetermined set of five signal peptides. Second, the analysis focused on a select number of the most effective clones for each construct. To comprehensively identify the optimal sequence and minimize the confounding effect of genomic integration position (position effect), future studies should utilize methods such as site-specific integration and high-throughput screening of large libraries of SP variants.

Also, as described in Peng et al.’s work [48], although SPs are generally universal across species, studies have shown that the same SP can result in significant differences in protein secretion when used in different hosts. Unfortunately, SignalP 6.0 offers only two options: Eukarya or Other, and analyzes only amino acid sequences. Therefore, factors such as gene silencing or mRNA stability are not taken into account. Furthermore, species specificity is not considered in frame of this empirical method.

In conclusion, our work not only validates the effectiveness of signal peptide swapping for enhancing glucoamylase production in *P. verruculosum* but also provides clear evidence that SP specificity, rather than gene copy number, is the dominant factor for secretion efficiency in this particular host. A promising direction for future research involves the creation and high-throughput screening of an expanded library of chimeric constructs, incorporating both homologous and synthetic signal peptides, to further maximize the yield of the target enzyme.

## 5. Conclusions

This study revealed that when using the technique of replacing various heterologous and homologous enzymes, homologous signal peptides demonstrate the best results. It should be emphasized that the selection of a pvGlaA SP from *P. verruculosum* proved to be the most effective strategy for the large-scale production of heterologous glucoamylase. A combination of a strong inducible *cbh1* promoter and a pvGlaA SP can significantly enhance the *P. verruculosum* expression platform for increasing the secretion of industrially important enzymes such as lipase, proteases, and other in-demand enzymes.

This approach can be used to construct other industrial strains of technical and food value, which is of great importance for industrial biotechnology, as it expands the methodological possibilities for obtaining producers of target enzymes. This technique can also be recommended for other strains of filamentous fungi.

## Figures and Tables

**Table 1:** Probabilities of SP cleavage from glucoamylase aaGlaA*.

SP	Enzyme	Microorganism	SP, Length, aa	Probability of Cleavage * [25]
aaGlaA	glucan1,4-α-glucosidase, GH15 EC number “3.2.1.3”	*A. awamori*	24	0.598458
CbhI	cellobiohydrolase I, GH7, EC number “3.2.1.176”	*P. verruculosum*	25	0.942205
pvGlaA	glucan1,4-α-glucosidase, GH15, EC number “3.2.1.3”	*P. verruculosum*	18	0.979223
BglI	β-glucosidase, GH3 EC number “3.2.1.21”	*A. niger*	19	0.971743
XylA	endo-1,4-β-xylanase GH10, EC number “3.2.1.8”	*P. canescens*	25	0.894761

*—see details in Supplementary Materials.

**Table 2:** Number of clones obtained during transformation.

Signal Peptide Contained in the Construct	Number of Clones
Bgl1 SP (β– glucosidase peptide from *A. niger*)	37
Cbh1 SP (cellobiohydrolase I peptide from *P. verruculosum*)	26
pvGlaA SP (glucoamylase A peptide from *P. verruculosum*)	34
XylA SP (xylanase A peptide from *P. canescens*)	34
aaGlaA SP (nature peptide glucoamylase A (from *A. awamori*)	33

**Table 3:** Screening of the recombinant clones in 24-well plates.

№ Clone	pvGlaA SP			№ Clone	XylA SP		
	Activity Glucoamilase (U/mL)	Concentration of Protein (mg/mL)	Specific Activity (U/mg)		Activity Glucoamilase (U/mL)	Concentration of Protein (mg/mL)	Specific Activity (U/mg)
1	46 ± 3 ^b^	2.4 ± 0.1 ^c^	18.9 ± 1.3	1	36 ± 3 ^a^	3.9 ± 0.2 ^a^	9.4 ± 0.7
4	45 ± 3 ^b^	2.8 ± 0.2 ^c^	15.9 ± 1.0	2	79 ± 6 ^c^	4.5 ± 0.3 ^b^	17.4 ± 1.3
6	26 ± 2 ^c^	2.0 ± 0.1 ^c^	13.3 ± 1.0	3	24 ± 2 ^c^	2.1 ± 0.1 ^c^	11.5 ± 0.8
8	53 ± 4 ^a^	1.8 ± 0.1 ^c^	30.0 ± 2.2	6	115 ± 8 ^c^	5.3 ± 0.3 ^c^	5.0 ± 0.3
9	54 ± 4 ^a^	4.1 ± 0.3 ^b^	13.4 ± 1.0	7	89 ± 6 ^c^	5.9 ± 0.4 ^c^	15 ± 1
18	59 ± 4 ^a^	2.4 ± 0.2 ^c^	24.8 ± 1.8	8	32 ± 2 ^c^	3.1 ± 0.2 ^c^	10.3 ± 0.7
19	101 ± 7 ^c^	3.3 ± 0.2 ^b^	30.9 ± 2.0	9	32 ± 2 ^c^	2.3 ± 0.2 ^c^	13.7 ± 0.9
21	60 ± 4 ^b^	2.5 ± 0.2 ^c^	24.5 ± 1.6	10	20.0 ± 1.4 ^c^	2.8 ± 0.2 ^c^	7.1 ± 0.4
27	27 ± 2 ^c^	4.0 ± 0.2 ^b^	6.7 ± 0.4	11	27.2 ± 1.8 ^c^	7.3 ± 0.5 ^c^	3.7 ± 0.2
28	33 ± 2 ^c^	3.4 ± 0.2 ^b^	9.5 ± 0.7	13	17 ± 1 ^c^	11.2 ± 0.7 ^c^	1.5 ± 0.1
29	58 ± 3 ^a^	5.3 ± 0.4 ^c^	10.9 ± 0.8	14	31 ± 2.1 ^c^	1.5 ± 0.08 ^c^	21.0 ± 1.4
30	57 ± 4 ^a^	4.8 ± 0.3 ^c^	11.8 ± 0.7	19	11.1 ± 0.7 ^c^	3.7 ± 0.2 ^b^	3.0 ± 0.2
32	66 ± 4 ^b^	6.2 ± 0.4 ^c^	10.6 ± 0.8	21	26.5 ± 1.8 ^c^	2.3 ± 0.2 ^c^	11.5 ± 0.7
33	91 ± 6 ^c^	6.2 ± 0.5 ^c^	14.8 ± 1.1	28	41 ± 3 ^a^	2.7 ± 0.2 ^c^	15.4 ± 1.0
№ clone	Bgl1 SP			№ clone	aaGlaA SP		
	Activity glucoamilase (U/mL)	Concentration of protein (mg/mL)	Specific activity (U/mg)		Activity glucoamilase (U/mL)	Concentration of protein (mg/mL)	Specific activity (U/mg)
2	29 ± 2 ^c^	3.9 ± 0.2 ^a^	7.7 ± 0.5	3	18 ± 1 ^c^	1.6 ± 0.1 ^c^	11.2 ± 0.9
3	10.5 ± 0.7 ^c^	2.1 ± 0.1 ^c^	5.1 ± 0.4	6	77 ± 7 ^c^	3.4 ± 0.3 ^b^	23.1 ± 1.7
4	13.6 ± 0.9 ^b^	4.0 ± 0.3 ^b^	3.4 ± 0.2	7	13 ± 1 ^c^	2.4 ± 0.2 ^a^	5.3 ± 0.3
5	14.5 ± 1.0 ^b^	3.6 ± 0.2 ^b^	4.0 ± 0.3	9	26 ± 2 ^c^	3.4 ± 0.3 ^b^	7.7 ± 0.7
6	14.5 ± 0.9 ^b^	5.9 ± 0.3 ^c^	2.5 ± 0.2	10	21 ± 2 ^c^	6.3 ± 0.5 ^c^	3.4 ± 0.2
7	40 ± 3 ^c^	5.0 ± 0.3 ^c^	8.1 ± 0.5	11	36 ± 2 ^a^	2.2 ± 0.2 ^c^	16.6 ± 1.4
9	11.0 ± 0.7 ^c^	1.4 ± 0.1 ^c^	7.8 ± 0.6	13	69 ± 5 ^c^	3.2 ± 0.2 ^a^	21 ± 2
10	12.0 ± 0.8 ^c^	4.7 ± 0.3 ^a^	2.5 ± 0.2	14	11.5 ± 0.6 ^c^	2.0 ± 0.1 ^c^	5.6 ± 0.4
12	29 ± 2 ^c^	7.1 ± 0.4 ^b^	4.1 ± 0.3	19	40 ± 3 ^b^	2.0 ± 0.1 ^c^	20 ± 2
13	11.8 ± 0.8 ^c^	2.1 ± 0.2 ^c^	5.7 ± 0.4	20	33 ± 2 ^a^	13 ± 1 ^c^	2.6 ± 0.2
14	13.9 ± 0.9 ^b^	2.5 ± 0.1 ^c^	5.7 ± 0.4	23	22 ± 2 ^b^	5.8 ± 0.5 ^b^	3.8 ± 0.3
18	10.7 ± 0.8 ^c^	2.5 ± 0.2 ^c^	4.3 ± 0.3	27	46 ± 4 ^c^	11.3 ± 0.8 ^a^	4.1 ± 0.3
25	32 ± 2 ^c^	10.1 ± 0.7 ^c^	3.2 ± 0.2	32	12 ± 1 ^c^	1.07 ± 0.07 ^c^	11.6 ± 0.9
30	12 ± 1 ^c^	8.2 ± 0.5 ^b^	1.5 ± 0.1				
№ clone	Cbh1 SP						
	Activity glucoamilase (U/mL)	Concentration of protein (mg/mL)	Specific activity (U/mg)				
3	18.1 ± 1.3 ^b^	3.4 ± 0.3 ^a^	5.4 ± 0.4				
8	14.6 ± 1.1 ^a^	2.7 ± 0.2 ^b^	5.4 ± 0.4				
10	13.5 ± 1.0 ^b^	2.8 ± 0.2 ^b^	4.8 ± 0.3				
12	12.4 ± 0.9 ^b^	5.7 ± 0.4 ^c^	2.1 ± 0.1				
15	20.2 ± 1.4 ^b^	2.4 ± 0.2 ^b^	8.4 ± 0.6				
18	15 ± 1 ^a^	3.3 ± 0.2 ^a^	4.5 ± 0.3				
19	13.0 ± 0.8 ^b^	3.1 ± 0.2 ^a^	4.3 ± 0.2				

(a) *p* (<0.1), (b) *p* (<0.05), (c) *p* (<0.01).

**Table 4:** Dynamics of accumulation of glucoamylase activity in 1.5 L fermenters for pvGlaA SP21, XylA SP2, aaGlaA SP6, and Bgl1 SP2.

Time of Cultivation		pvGlaA SP21	
	Activity of aaGlaA* (U/mL)	Protein Concentration (mg/mL)	Specific Activity (U/mg)
48 h	91 ± 8.1 ^c^	3.3 ± 0.5 ^c^	28 ± 1.4
72 h	330± 29 ^c^	9.7 ± 0.6 ^c^	34 ± 2.7
96 h	820 ± 52 ^c^	17 ± 1.3 ^c^	49 ± 3.8
120 h	1110 ± 76 ^c^	27 ± 2.3 ^a^	41 ± 2.3
144 h	2400 ± 180 ^c^	26 ± 2.3 ^a^	92 ± 6.7
		XylA SP2	
	Activity of aaGlaA* (U/mL)	Protein concentration (mg/mL)	Specific activity (U/mg)
48 h	150 ± 10 ^c^	2.2 ± 0.13 ^c^	68 ± 5
72 h	430 ± 35 ^c^	13.0 ± 0.8 ^c^	32 ± 2.2
96 h	840 ± 60 ^c^	23 ± 1.5 ^b^	36 ± 2.4
120 h	1330 ± 90 ^c^	29 ± 1.9 ^a^	45 ± 3.1
144 h	1750 ± 120 ^b^	27 ± 1.8 ^a^	64 ± 5
		aaGlaA SP6	
	Activity of aaGlaA* (U/mL)	Protein concentration (mg/mL)	Specific activity (U/mg)
48 h	35 ± 3.2 ^c^	1.3 ± 0.2 ^c^	27 ± 4
72 h	170 ± 15 ^c^	15 ± 1.0 ^c^	11.1± 0.7
96 h	450 ± 27 ^c^	23 ± 1.6 ^c^	20 ± 2
120 h	740 ± 47 ^c^	30 ± 2.1 ^b^	25 ± 2.1
144 h	870 ± 62 ^b^	30 ± 2.0 ^a^	29 ± 2.9
		Bgl1 SP2	
	Activity of aaGlaA* (U/mL)	Protein concentration (mg/mL)	Specific activity (U/mg)
48 h	32 ± 3,1 ^c^	0.31 ± 0.02 ^c^	103 ± 12
72 h	190 ± 14 ^c^	14.9 ± 0.96 ^c^	12.5 ± 0.7
96 h	490 ± 36 ^c^	24 ± 1.5 ^c^	20.6 ± 1.7
120 h	770 ± 56 ^c^	28 ± 2.3 ^b^	27 ± 2.2
144 h	880 ± 59 ^b^	29 ± 2.2 ^a^	30 ± 2.6

(a) *p* (<0.1), (b) *p* (<0.05), (c) *p* (<0.01).

**Table 5:** Glucoamylase content and glucoamylase activity in the dry enzymatic preparations (U per g).

EP	Activity of aaGlaA* (U/g)	aaGlaA* Content (%)
aaGlaA SP 6	19,000 ± 1100	43.6
pvGlaA SP 21	33,300 ± 900	77.4
XylA SP 2	28,600 ± 800	74.1
Bgl1 SP 2	23,500 ± 800	59.8

## Data Availability

The authors declare that the data supporting the findings of this study are available within the main text of the manuscript. Raw data is available from the corresponding authors upon reasonable request.

## Supplementary Materials

The following supporting information can be downloaded at https://www.mdpi.com/article/10.3390/jof12020085/s1, Figure S1: Nucleotide sequences and corresponding amino acid sequences for *aaglaA** in the filamentous fungus *A. awamori*; Table S1: Primers used in this study; Figure S2: Nucleotide sequences and corresponding amino acid sequences for SPs; Figure S3: Prediction of signal peptide structures and probabilities of cleavage; Table S2: Possible masses of signal peptide fragments calculated during theoretical cleavage of the aaGlaA sequence; Figure S4: SDS-PAGE of selected recombinants; Table S3: Peptides corresponding to the N- and C-termini of the aaGlaA amino acid chain; Figure S5: Chromatogramm of enzymatic preparations after ion exchange chromatography; Table S4: Protein concentrations and glucoamylase activity toward starch obtained in the flasks; Figure S6: Quantification Cq results to determine the number of copies of the *aaglaA* gene; Figure S7: Gene expression results - scatter plot to determine the number of copies of the *aaglaA* gene; Figure S8: Quantification Cq results for the determination of *aaglaA* transcription level; Figure S9: Gene Expression Results for aaglaA* gene in aaGlaA SP6, Bgl1 SP2, pvGlaA SP21 and XylA SP2 strains.
