# Supplementary material for: Effect of Different Signal Peptides on the Expression of Glucoamylase from Aspergillus awamori in the Filamentous Fungus Penicillium verruculosum
Source: J Fungi (Basel). 2026 Jan 27;12(2):85. doi: 10.3390/jof12020085 (PMC12942012; doi:10.3390/jof12020085)
Supplement: Supplementary file 1 [file jof-12-00085-s001.zip › jof-4059841-supplementary.pdf]

Table S1. Primers used in this study

Gene	Primer name	5`-3`	Use
cbh1	SScbhF	GTACAAGAGCGCCCTCATCTTGGGTTCCCTGCTGGCAACAGCTGGTGCTGCGACCTTGGATTCGTGGTT	Change SP
	SScbhR	AGATGAGGGCGCTCTTGTACATGTTGAAAGAGTTCAAGGCAGACATTGTGTCGGTTCTCTGTTTGAG	
bgl1	SSbglF	AGGCGGTGGCTCTGACTGCCGTCTCGCTGGCCAGCGCTGCGACCTTGGATTCGTGGTT	
	SSbglR	AGTCAGAGCCACCGCCTCGATCAAAGTGAACCTCATTGTGTCGGTTCTCTGTTTGAG	
pvglaA	SSglaF	GTTGGCCGCACTAAACCTGCTGACCTTGGTGCAGGGAGCGACCTTGGATTCGTGGTT	
	SSglaR	AGGTTTAGTGCGGCCAACGCAGTGGTCACAACCATTGTGTCGGTTCTCTGTTTGAG	
xylA	SSxylF	GCTTCTTTTCGCCGGTCAGGCTCTTTCCGGACCTGTCGACTCCCGAGCGACCTTGGATTCGTGGTT	
	SSxylR	GACCGGCGAAAAGAAGCGCCAGAGCAGCAGTCTTGAGTTGAACCATTGTGTCGGTTCTCTGTTTGAG	
aaglaA	N181Q-fwd	GAAGAAGTCCAAGGCTCGTCTTTC	Introduction of N181Q mutation in *aaglaA*
	N181Q-rev	GAAAGACGAGCCTTGGACTTCTTC	
aaglaA	aaGlaLICF	CAAACAGAAGCAACCGACACAATTGTCGTTCCGATCTCTTCTCGCCCTG	Cloning of *aaglaA* gene
	aaGlaLICR	GAGGAGAAGCCCGGTCTACCGCCAGGTGTCAGTCACCG	
aaglaA	GlaA_Front	CATCTACCTGGTCGGATCAATCTCAC	qPCR
	GlaA_Reverse	GGCAAAGTCACAGTGACGTACCAGAG	
actA	PVACTQF1	ACAAGAAATCCAGACCGCTTCCC	
	PVACTQR1	TTTCGAGACCGATGACGGAAGG	

M S F R S L L A L S G L V C S G L A S V I S K R A T L D S W L S N E ·

1 ATGTCGTTCCGATCTCTTCTCGCCCTGAGCGGCCTCGTCTGCTCGGGGTTGGCTAGTGTTATTTCCAAGCGCGCGACCTTGGATTCGTGGTTGAGCAACG

· A T V A R T A I L N N I G A D G A W V S G A D S G I V V A S P S T ·

101 AAGCGACCGTGGCTCGTACTGCCATCCTGAATAACATCGGGGCGGACGGTGCTTGGGTGTCGGGCGCCGACTCTGGCATTGTCGTTGCTAGTCCCAGCAC

· D N P D

201 GGATAACCCGGACT*GTATGTTTTCGAGCTGAGAGCACGTCTAATATATCATGAGCGTGATTTGATCGTTGATTGATTGATGCTGATTGATGTGTTTGTTA*

· Y F Y T W T R D S G L V I K S L V D L F R N G D T S L L S T I E

301 *TTGAA*ATTTCTACACCTGGACTCGCGACTCTGGTCTCGTCATCAAGTCCCTCGTCGATCTCTTCCGAAACGGAGATACCAGTCTGCTCTCCACCATTGAG

H Y I S S Q A I I Q G V S N P S G D L S S G G L G E P K F N V D E T ·

401 CACTACATCTCCTCTCAAGCGATCATCCAGGGTGTCAGTAACCCCTCTGGTGATCTGTCGAGCGGTGGTCTCGGTGAGCCCAAGTTCAATGTCGATGAGA

· A Y T G S W G R P Q R D G P A L R A T A M I D F G N W L I

501 CTGCCTACACTGGGTCTTGGGGACGGCCGCAGCGAGATGGTCCGGCTCTGAGAGCAACTGCTATGATCGACTTCGGGAATTGGTTGATT*GTACGTCCTCC*

D N G Y T S V A T D I V W P L V R ·

601 *ACTTCCCCCCTTGCGTGTGATTTGCGACGTATGTAGCTGACTAGTAGTCAG*GATAATGGCTACACCAGTGTCGCCACGGATATTGTTTGGCCCCTCGTTA

· N D L S Y V A Q Y W N Q T G Y

701 GAAACGACCTGTCGTATGTGGCGCAGTACTGGAACCAGACGGGATATG*GTGTGTTTGATCGATCGGTATTCGAGGGTGTGCATCGGAGCTAACTCGCGGT*

D L W E E V **Q** G S S F F T I A V Q H R A L V E G S A F A T A V G

801 *CGCAG*ATCTCTGGGAAGAAGTC**C**A**A**GGCTCGTCTTTCTTCACCATTGCTGTGCAGCACCGCGCCCTTGTCGAGGGTAGTGCCTTCGCGACGGCCGTCGGC

S S C S W C D S Q A P Q I L C Y L Q S F W T G S Y I L A N F D S S R ·

901 TCGTCCTGCTCCTGGTGTGATTCGCAGGCACCTCAGATTCTCTGCTACCTGCAGTCCTTCTGGACCGGCAGCTACATTCTGGCCAACTTCGATAGCAGCC

· S G K D A N T L L G S I H T F D P E A G C D D S T F Q P C S P R A ·

1001 GTTCCGGCAAGGACGCGAACACCCTCCTGGGAAGCATCCATACCTTTGATCCTGAGGCCGGATGTGACGACTCGACCTTCCAGCCCTGCTCCCCGCGTGC

· L A N H K E V V D S F R S I Y T I N D G L S D S E A V A V G R Y P

1101 GCTCGCCAACCACAAGGAGGTTGTAGACTCTTTCCGCTCGATTTATACCATCAACGACGGTCTCAGTGACAGTGAAGCTGTTGCGGTCGGTCGGTACCCC

E D S Y Y N G N P W F L C T L A A A E Q L Y D A L Y Q W D K Q G E L ·

1201 GAGGATTCGTACTACAACGGCAACCCGTGGTTCCTGTGCACCTTGGCTGCCGCGGAGCAGCTGTACGACGCTTTGTACCAATGGGACAAGCAGGGAGAGT

· E I T D V S L D F F Q A L Y S S A A T G T Y S S S S S T Y S S I V ·

1301 TGGAGATTACCGACGTGTCGCTGGACTTCTTCCAGGCTCTGTACAGCAGTGCTGCCACAGGCACTTACTCTTCGTCCAGCTCGACCTATAGCAGCATTGT

· D A V K T F A D G F V S I V

1401 GGATGCTGTGAAGACCTTTGCCGATGGCTTCGTTTCTATTGTT*GTAAGTCTACACCAGACGTGTGCTCGTATCTACCTGGGATGTGTACTAACAGGATTA*

E T H A A S N G S L S E Q F D K S S G E E L S A R D L T W S Y A A

1501 *G*GAAACTCACGCCGCAAGCAATGGCTCTTTGTCCGAGCAATTTGACAAGTCTAGTGGCGAGGAGCTTTCTGCTCGTGATTTGACCTGGTCTTACGCTGCT

L L T A N N R R N S V V P P S W G E T S A S S V P G S C V A T S A S ·

1601 CTGCTGACTGCCAACAACCGCCGCAACTCTGTCGTACCTCCCTCCTGGGGCGAGACTTCTGCGAGCAGCGTGCCCGGATCCTGTGTGGCCACCTCTGCCT

· G T Y S S V T V T S W P S I V A T G G T T T T A T T T G S G G V ·

1701 CTGGTACCTACAGCAGTGTGACTGTCACCTCGTGGCCAAGTATCGTCGCTACCGGCGGCACCACTACTACGACTGCTACCACCACTGGATCCGGCGGCGT

· T S T S K T T T T A S K T S T T T S S T S C T T P T A V A V T F D

```
1801     GACCTCGACCAGCAAGACCACTACGACTGCTAGTAAGACCAGTACCACTACATCCTCCACTTCCTGCACCACTCCCACCGCCGTGGCCGTGACTTTTGAT
          L  T  A  T  T  T  Y  G  E  S  I  Y  L  V  G  S  I  S  Q  L  G  D  W  D  T  S  D  G  V  A  L  S  A  D ·
1901     CTGACGGCCACCACCACCTACGGAGAGAGCATCTACCTGGTCGGATCAATCTCACAGCTCGGCGACTGGGACACCAGTGACGGCGTGGCTCTGAGCGCTG
         ·  K  Y  T  S  S  N  P  L  W  Y  V  T  V  T  L  P  A  G  E  S  F  E  Y  K  F  I  R  V  E  S  D  D  T ·
2001     ACAAATACACTTCCAGCAACCCACTCTGGTACGTCACTGTGACTTTGCCGGCTGGAGAGTCGTTCGAGTACAAGTTCATCCGCGTTGAGAGCGATGACAC
         · V  E  W  E  S  D  P  N  R  E  Y  T  V  P  Q  T  C  G  X  S  T  V  T  V  T  D  T  W  R  *
2101     CGTGGAGTGGGAGAGTGACCCGAACCGGGAATACACCGTTCCTCAGACGTGCGGAGANTCGACCGTGACGGTGACTGACACCTGGCGGTAG
```

Figure S1. Nucleotide sequences and corresponding amino acid sequences for *aaglaA** in the filamentous fungus *A. awamori*. Putative intron sequences are underlined. The SP sequence and N181Q mutation are bold in green boxes.

```
aaglaA
           M  S  F  R  S  L  L  A  L  S  G  L  V  C  S  G  L  A  S  V  I  S  K  R
          ATGTCGTTCCGATCTCTTCTCGCCCTGAGCGGCCTCGTCTGCTCGGGGTTGGCTAGTGTTATTTCCAAGCGC

cbh1
           M  S  A  L  N  S  F  N  M  Y  K  S  A  L  I  L  G  S  L  L  A  T  A  G  A
          ATGTCTGCCTTGAACTCTTTCAACATGTACAAGAGCGCCCTCATCTTGGGTTCCCTGCTGGCAACAGCTGGTGCT

pvglaA
           M  V  V  T  T  A  L  A  A  L  N  L  L  T  L  V  Q  G
          ATGGTTGTGACCACTGCGTTGGCCGCACTAAACCTGCTGACCTTGGTGCAGGGA

bgl1
           M  R  F  T  L  I  E  A  V  A  L  T  A  V  S  L  A  S  A
          ATGAGGTTCACTTTGATCGAGGCGGTGGCTCTGACTGCCGTCTCGCTGGCCAGCGCT

xylA
           M  V  Q  L  K  T  A  A  L  A  L  L  F  A  G  Q  A  L  S  G  P  V  D  S  R
          ATGGTTCAACTCAAGACTGCTGCTCTGGCGCTTCTTTTCGCCGGTCAGGCTCTTTCCGGACCTGTCGACTCCCGA
```

Figure S2. Nucleotide sequences and corresponding amino acid sequences for SPs.

A

aaGlaA*

Prediction: Signal Peptide (Sec/SPI)
Cleavage site between pos. 24 and 25. Probability 0.598458

Protein type	Other	Signal Peptide (Sec/SPI)
Likelihood	0.0162	0.9838

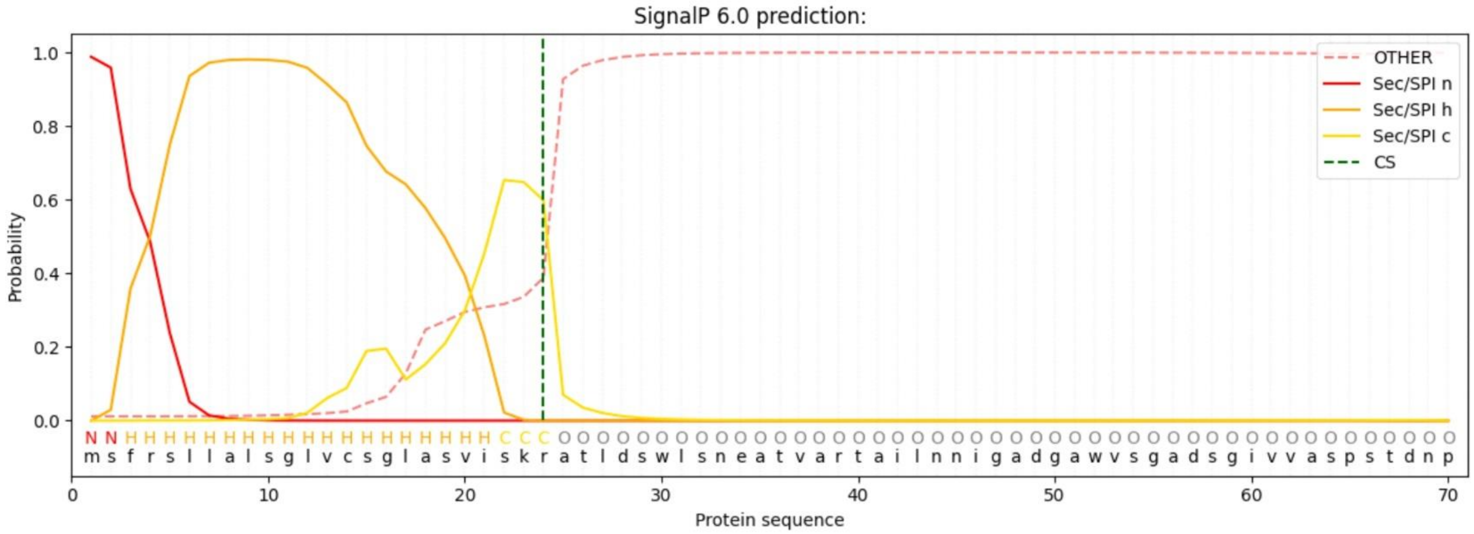

B

CbhI_SP::aaGlaA*

Prediction: Signal Peptide (Sec/SPI)
Cleavage site between pos. 25 and 26. Probability 0.942205

Protein type	Other	Signal Peptide (Sec/SPI)
Likelihood	0.0004	0.9995

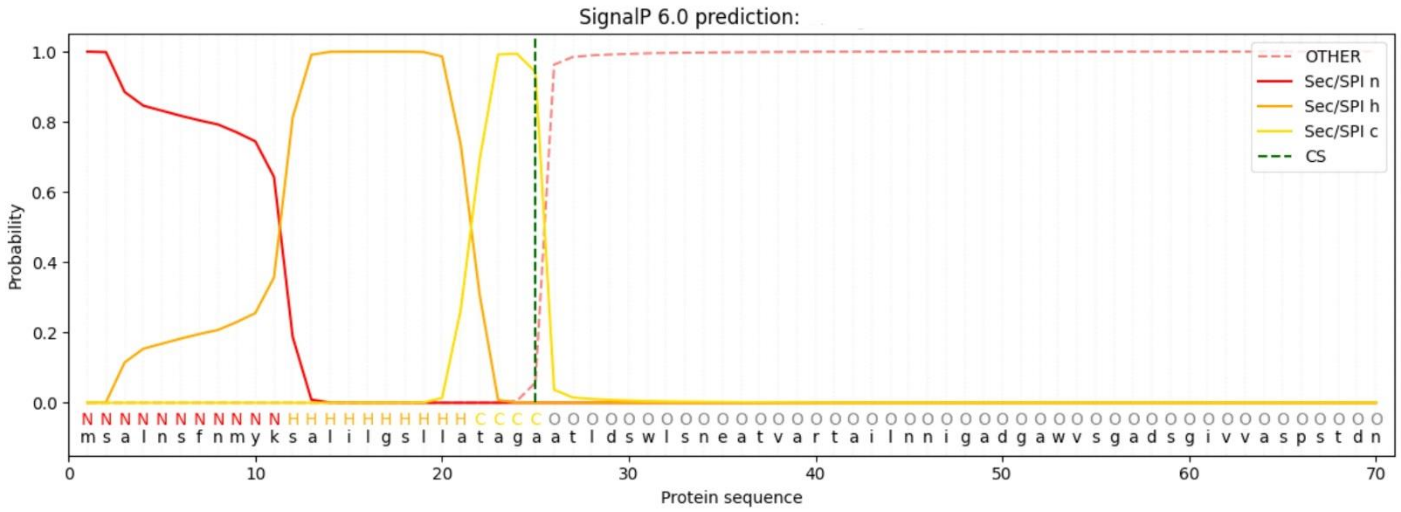

C

pvGlaA_SP::aaGlaA*

Prediction: Signal Peptide (Sec/SPI)
Cleavage site between pos. 18 and 19. Probability 0.979223

Protein type	Other	Signal Peptide (Sec/SPI)
Likelihood	0.0003	0.9997

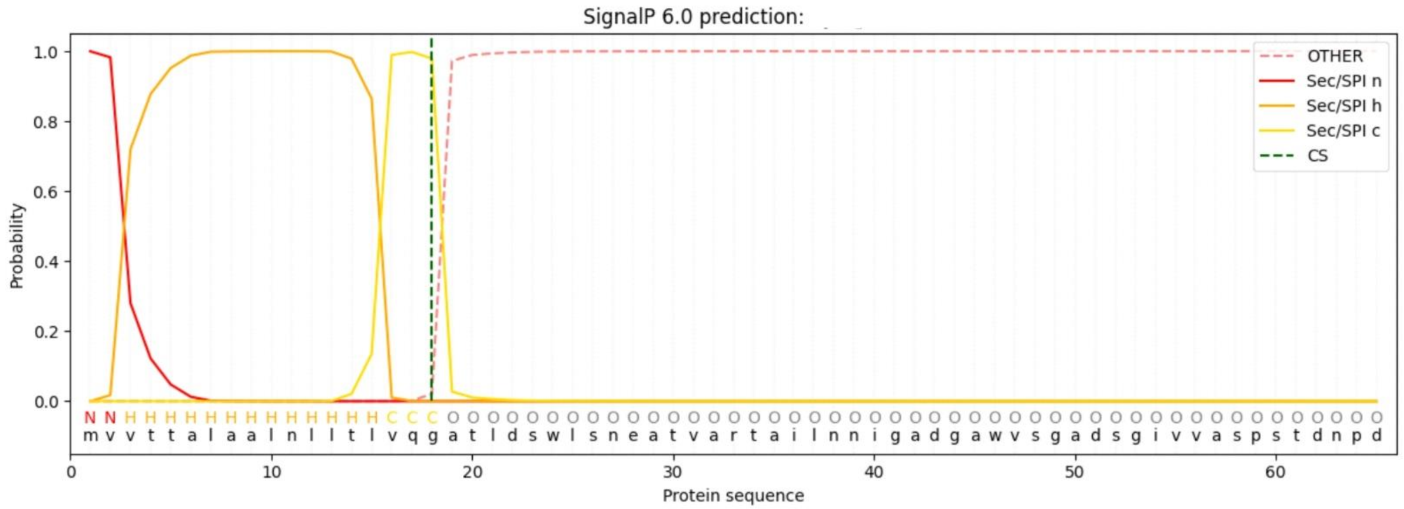

D

Bgl1_SP::aaGlaA*

Prediction: Signal Peptide (Sec/SPI)
Cleavage site between pos. 19 and 20. Probability 0.971743

Protein type	Other	Signal Peptide (Sec/SPI)
Likelihood	0.0003	0.9997

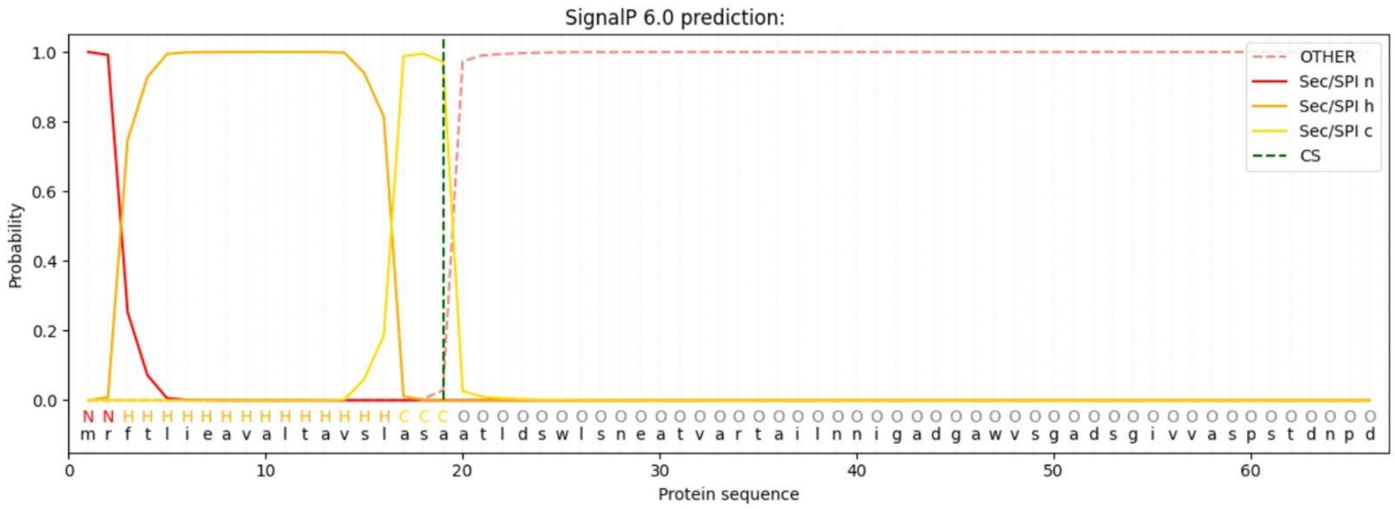

E

XylA_SP::aaGlaA*

Prediction: Signal Peptide (Sec/SPI)
Cleavage site between pos. 25 and 26. Probability 0.894761

Protein type	Other	Signal Peptide (Sec/SPI)
Likelihood	0.0002	0.9997

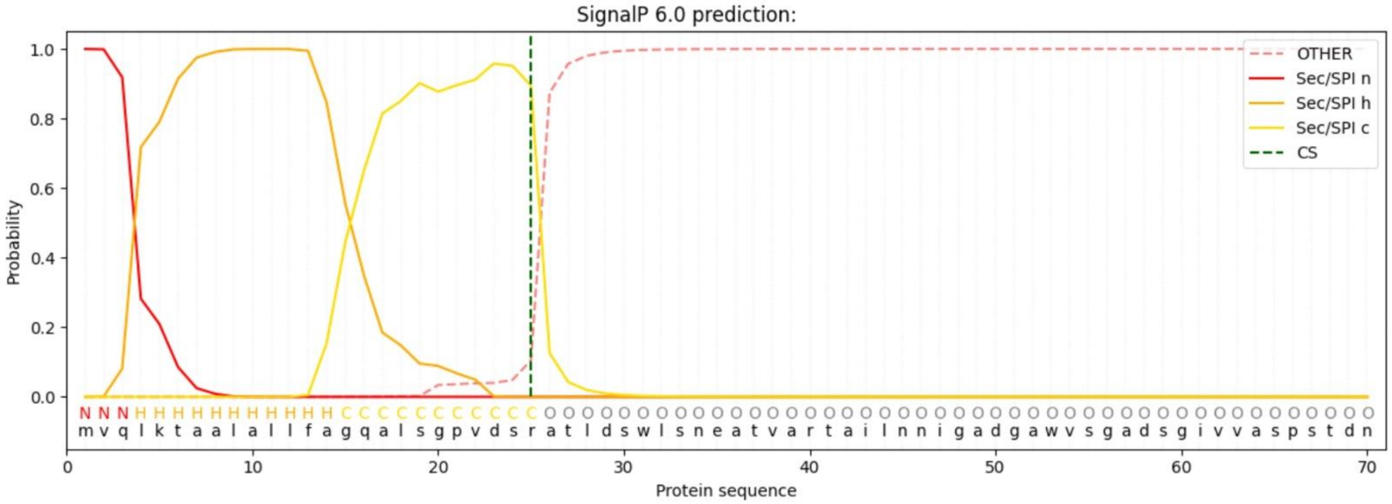

F

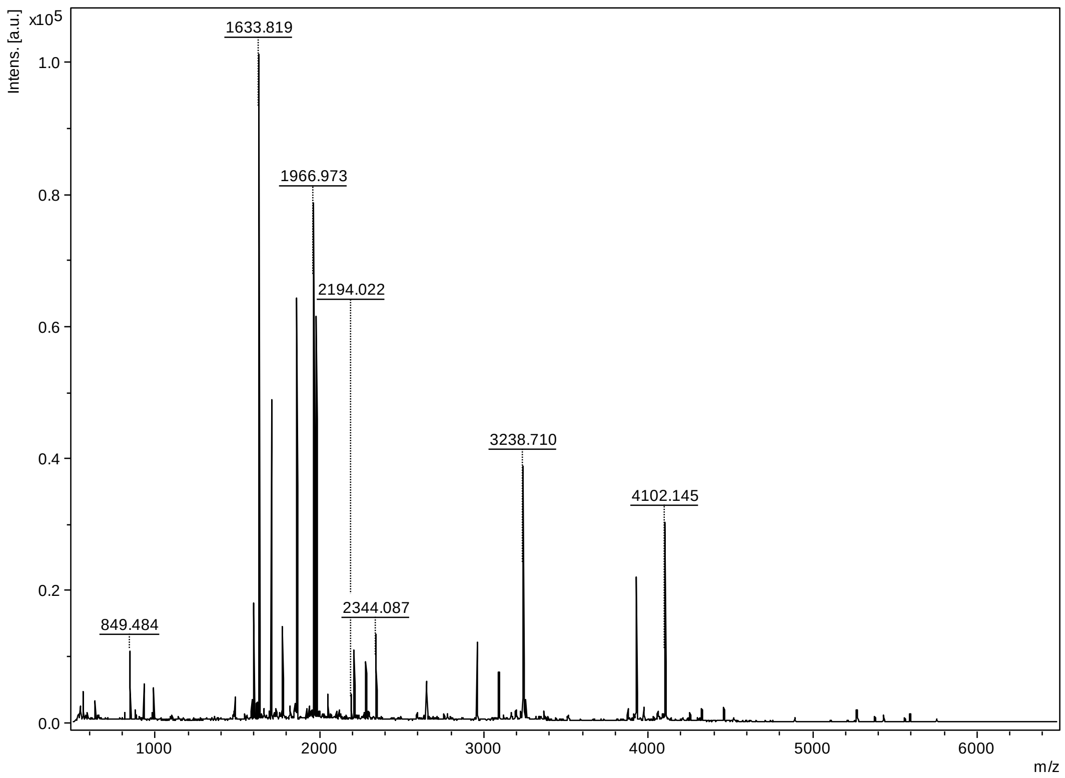

Figure S3 – Prediction of signal peptide structures and probabilities of cleavage for: **A**- aaGlaA SP, **B**- CbhI SP, **C**- pvGlaA SP, **D**- BglI SP, **E**- XylA SP. The data was obtained using the Program SignalP 6.0 (https://services.healthtech.dtu.dk/services/SignalP-6.0/). **F**- Mass spectrometric analysis of the amino acid sequence of aaGlaA. The masses of the peaks indicated in the figure are described in **Table S2** and **Table S3**.

Table S2 – Possible masses of signal peptide fragments calculated during theoretical cleavage of the aaGlaA sequence (The online service PeptideMass was used to calculate the masses of peptides https://web.expasy.org/peptide_mass/)

Mass	Position	Missed cleavages	Artif. modification(s)	Mass with modifications	Peptide sequence
540.260	1-4	0	MSO: 1	556.255	MSFR
2339.283	1-23	1	Cys_PAM: 14	2410.320	MSFRSLLALSGLVCSGLASV ISK
			MSO: 1	2355.277	
2495.384	1-24	2	Cys_PAM: 14	2566.421	MSFRSLLALSGLVCSGLASV ISKR
			MSO: 1	2511.379	
4110.184	1-39	3	Cys_PAM: 14	4181.221	MSFRSLLALSGLVCSGLASV ISKRATLDSWLSNEATVAR
			MSO: 1	4126.179	
1818.040	5-23	0	Cys_PAM: 14	1889.078	SLLALSGLVCSGLASVISK
1974.142	5-24	1	Cys_PAM: 14	2045.179	SLLALSGLVCSGLASVISKR
3588.942	5-39	2	Cys_PAM: 14	3659.979	SLLALSGLVCSGLASVISKR ATLDSWLSNEATVAR
1789.919	24-39	1			RATLDSWLSNEATVAR
5872.848	24-78	2			RATLDSWLSNEATVARTAIL NNIGADGAWVSGADSGIVVA SPSTDNPDYFYTWTR

Table S3 - Peptides corresponding to the N- and C-termini of the aaGlaA amino acid chain were detected. The peak with m/z 1633.819 (see **Figure S3 F**) corresponds to peptide 25-39, and the peak with m/z 2344.087 (see Figure **S3 F**) corresponds to peptide 621-640 (The FindPept online service was used to identify peaks https://web.expasy.org/findpept/).

Experimental mass	Theoretical mass	Δmass (daltons)	Peptide	Position	Modifications	Missed cleavages
849.484	849.483	-0.001	(K)/SLVDLFR/(N)	86-92		0
1485.796	1485.781	-0.015	(R)/ALANHKEVVDSFR/(S)	297-309		1
1633.819	1633.818	-0.001	(R)/ATLDSWLSNEATVAR/(T)	25-39		0
1864.965	1864.966	0.001	(R)/DLTWSYAALLTANNRR/(N)	437-452		1
1966.973	1966.972	-0.001	(R)/SIYTINDGLSDSEAVAVGR/(Y)	310-328		0
1983.935	1983.931	-0.004	(K)/FNVDETAYTGSWGRPQR/(D)	132-148		0
1999.952	1999.926	-0.026	(K)/FNVDETAYTGS<u>W</u>GRPQR/(D)	132-148	TPO	0
2194.022	2194.005	-0.017	(K)/FIRVESDDTVEWESDPNR/(E)	603-620		1
2344.087	2344.076	-0.010	(R)/EYTVPQTCGESTVTVTDTWR	621-640	CYS_PAM	0
3238.710	3238.624	-0.085	(R)/ATAMIDFGNWLIDNGYTSVA TDIVWPLVR/(N)	155-183		0
3254.709	3254.619	-0.090	(R)/ATAMIDFGN<u>W</u>LIDNGYTSVA TDIVWPLVR/(N)	155-183	TPO	0
3254.709	3254.619	-0.090	(R)/ATA<u>M</u>IDFGNWLIDNGYTSVA TDIVWPLVR/(N)	155-183	MSO	0
3254.709	3254.619	-0.090	(R)/ATAMIDFGNWLIDNGYTSVA TDIV<u>W</u>PLVR/(N)	155-183	TPO	0
3929.122	3928.941	-0.181	(R)/NGDTSLLSTIEHYISSQAII QGVSNPSGDLSSGGLGEPK/(F)	93-131		0
4102.145	4101.946	-0.198	(R)/TAILNNIGADGAWVSGADSG IVVASPSTDNPDYFYTWTR/(D)	40-78		0

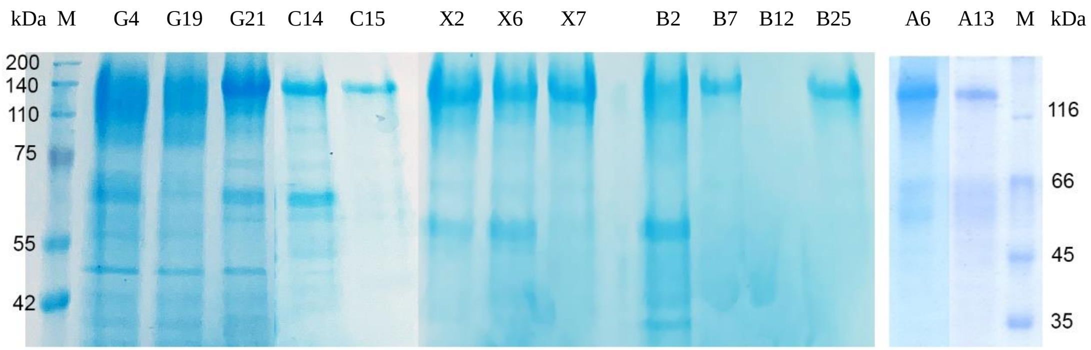

Figure S4 – SDS-PAGE of selected recombinants. M – protein markers, in kDa, k- host strain (G4, G19, G21– clones 4, 19, 21 series pvGlaA SP; C14, C15 – clones 14 and 15 series Cbh1 SP; X2, X6, X7 – clones 2, 6, 7 series XylA SP; B2, B7, B12, B25 – clones 2, 7, 12, 25 series Bgl1 SP; A6, A13 – clones 6, 13 series aaGlaA SP)

Table S4- Protein concentrations and glucoamylase activity toward starch obtained in the flasks for recombinant strains of the series pvGlaA SP, XylA SP, Cbh1 SP, Bgl1 SP, aaGlaA SP and host strain

№ clones	Protein conc (mg/ml)	Glucoamylase[1] activity (U/ml)	CMCase[2] activity (U/ml)	Xylanase[2] activity (U/ml)	Avicelase[3] activity (U/ml)	Specific activity of glucoamylase (U/mg)
pvGlaA SP 4	14,5 ± 0,6	330 ± 18	24 ± 1,6	290 ± 19	8,2 ± 0,53	23 ± 1,6
pvGlaA SP 19	10,2 ± 0,7	260 ± 18	24 ± 1,6	310 ± 11	5,1 ± 0,29	26 ± 1,8
**pvGlaA SP 21**	**8,0 ± 0,5**	**270 ± 12**	**6,9 ± 0,42**	**200 ± 15**	**2,8 ± 0,19**	**33 ± 1,3**
Cbh1 SP 14	6,0 ± 0,4	84 ± 5,8	11,3 ± 0,64	127 ± 8,4	6,8 ± 0,41	13,9 ± 0,96
Cbh1 SP 15	5,2 ± 0,4	70 ± 4,6	1,90 ± 0,09	126 ± 8,6	nd	13,4 ± 0,9
**XylA SP 2**	**10,2 ± 0,6**	**330 ± 22**	**15 ± 1,1**	**178 ± 2,5**	**5,7 ± 0,36**	**33 ± 2,1**
XylA SP 6	7,3 ± 0,4	100 ± 7	17,2 ± 0,8	190 ± 13	6,5 ± 0,42	14,0 ± 1,0
XylA SP 7	6,7 ± 0,4	135 ± 9	5,0 ± 0,43	138 ± 9,0	1,6 ± 0,13	20 ± 1,4
**Bgl1 SP 2**	**5,3 ± 0,3**	**190 ± 10**	**28 ± 1,4**	**278 ± 9,4**	**6,0 ± 0,24**	**36 ± 2,5**
Bgl1 SP 7	12,8 ± 0,4	63 ± 4	4,4 ± 0,25	113 ± 7,8	nd	4,9 ± 0,33
Bgl1 SP 12	4,0 ± 0,4	5,3 ± 0,32	4,5 ± 0,21	77 ± 5,2	1,9 ± 0,11	1,3 ± 0,11
Bgl1 SP 25	6,1 ± 0,5	102 ± 7	3,7 ± 0,22	128 ± 8,5	nd	17,1 ± 1,0
**aaGlaA SP 6**	**8,6 ± 0,8**	**240 ± 16**	**15 ± 1,1**	**260 ± 14**	**2,0 ± 0,11**	**28 ± 2,1**
aaGlaA SP 13	7,3 ± 0,5	180 ± 12	8,1 ± 0,45	230 ± 21	1,32 ± 0,09	24 ± 1,8
Control (host strain)	9,6 ± 0,6	4,3 ± 0,1	2,7 ± 0,11	83 ± 6,4	1,27 ± 0,09	0,45 ± 0,01

30 °C, pH 4.7.; [2] 50 °C, pH 5.0.; [3] 40 °C, pH 5.0.

A

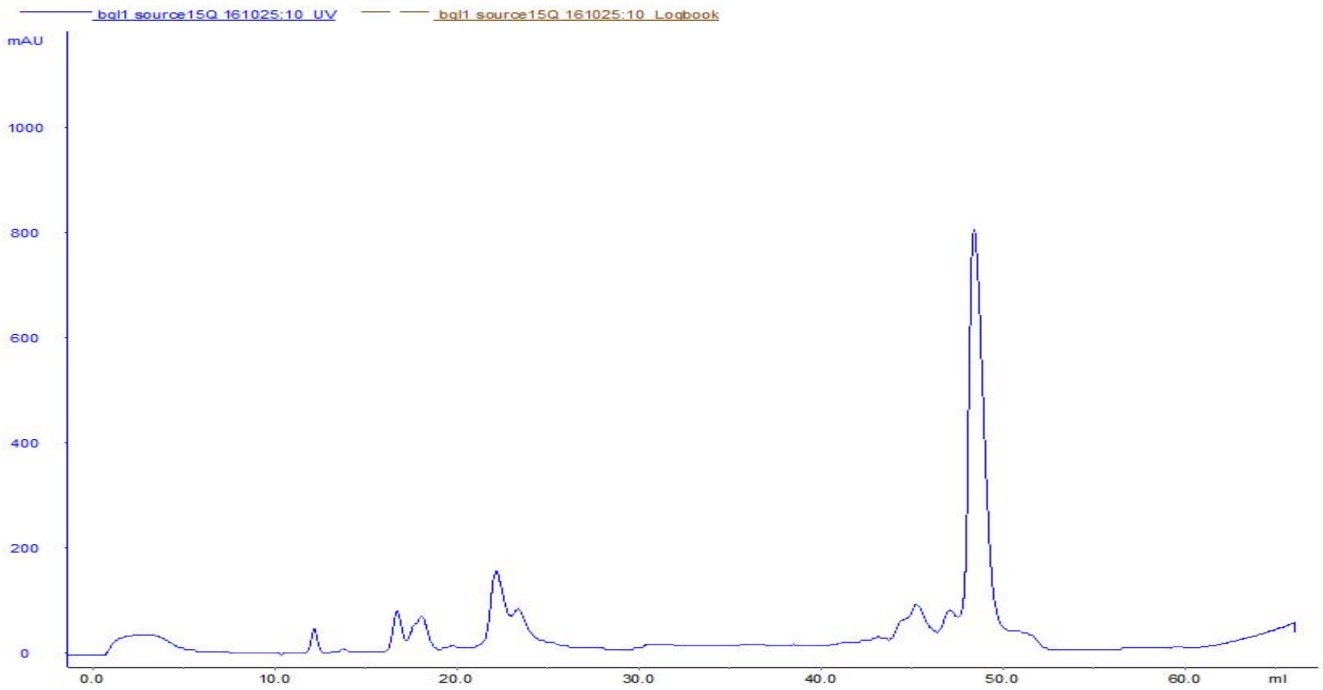

bgl1 source15Q 161025:10_UV
bgl1 source15Q 161025:10_Logbook
mAU
1000
800
600
400
200
0
0.0
10.0
20.0
30.0
40.0
50.0
60.0
ml

B

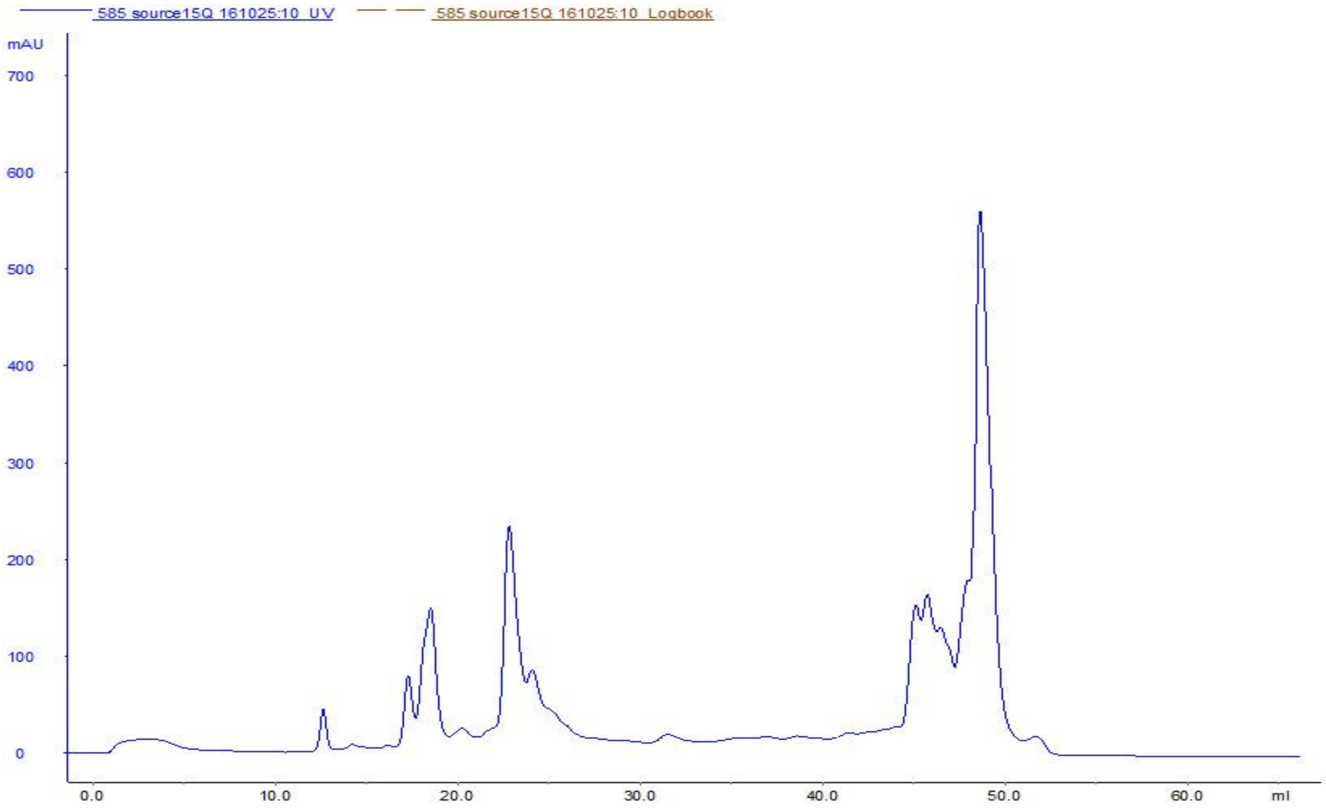

585 source15Q 161025:10_UV
585 source15Q 161025:10_Logbook
mAU
700
600
500
400
300
200
100
0
0.0
10.0
20.0
30.0
40.0
50.0
60.0
ml

C

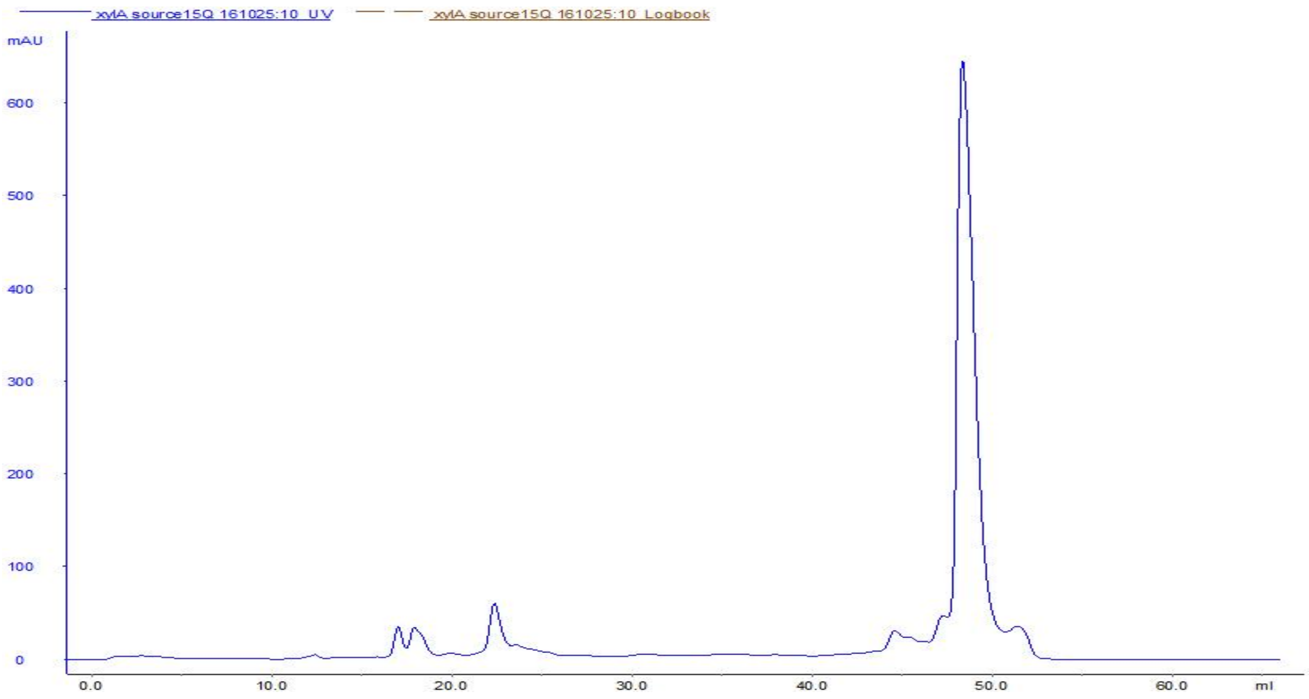
xylA source15Q 161025:10_UV
xylA source15Q 161025:10_Logbook
mAU
600
500
400
300
200
100
0
0.0
10.0
20.0
30.0
40.0
50.0
60.0
ml

D

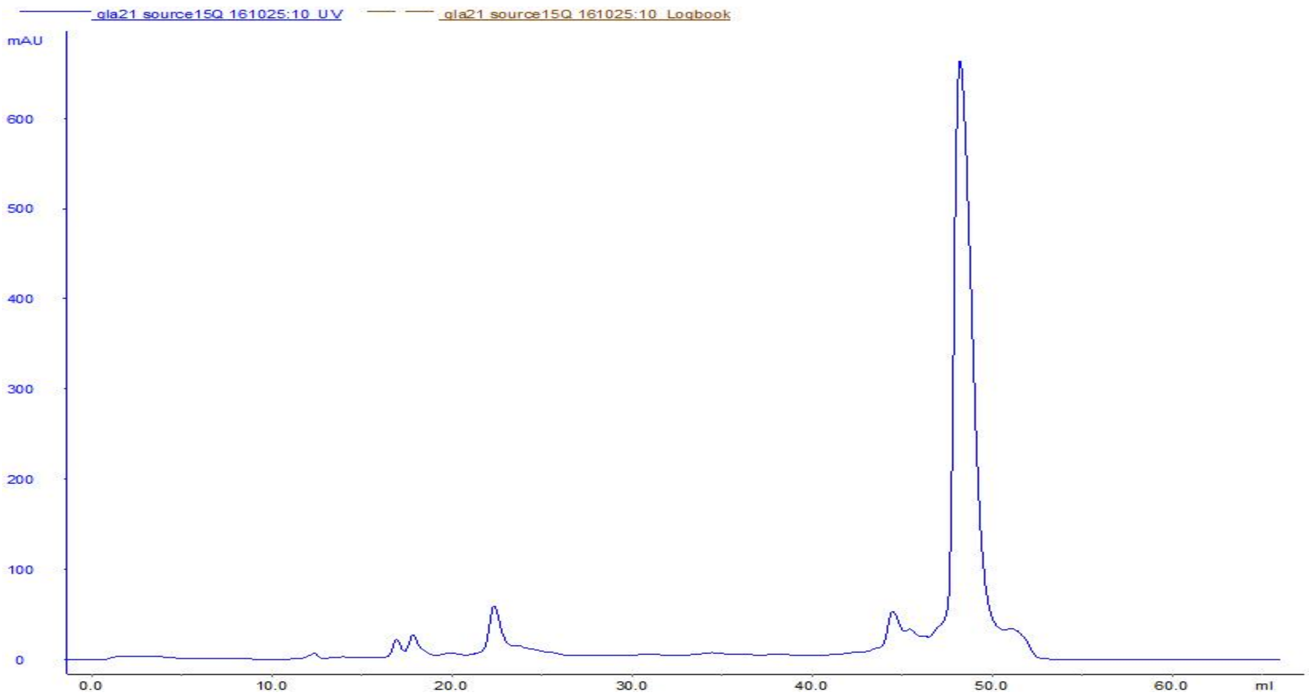

Figure S5 - Chromatogram of enzymatic preparations after ion exchange chromatography a) Bgl1 SP 2 b) aaGlaA SP 6 c) XylA SP 2 d) pvGlaA SP 21. The retention volume of GA peak is 48,2-48.3 ml.

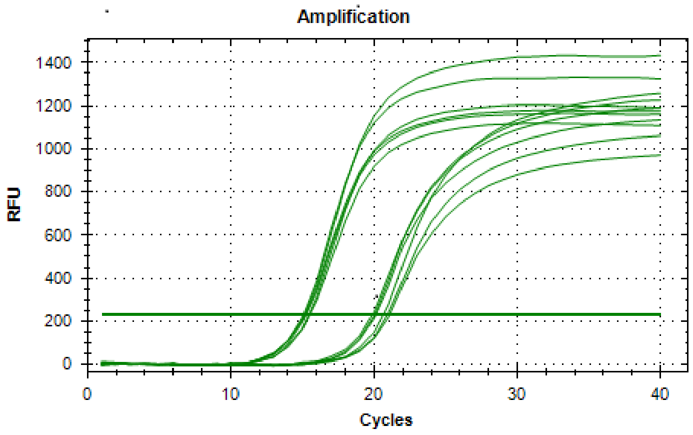

Well	Fluor	Target	Content	Sample	Cq	Cq Mean	Cq Std. Dev
E02	SYBR	actA	Unkn-02	pvGlaA SP21	20.14	20.04	0.117
F02	SYBR	actA	Unkn-02	pvGlaA SP21	20.06	20.04	0.117
G02	SYBR	actA	Unkn-02	pvGlaA SP21	19.91	20.04	0.117
E04	SYBR	aaglaA	Unkn-06	pvGlaA SP21	15.27	15.20	0.089
F04	SYBR	aaglaA	Unkn-06	pvGlaA SP21	15.22	15.20	0.089
G04	SYBR	aaglaA	Unkn-06	pvGlaA SP21	15.10	15.20	0.089
B05	SYBR	actA	Unkn-07	XylA SP2	21.12	20.91	0.228
C05	SYBR	actA	Unkn-07	XylA SP2	20.67	20.91	0.228
D05	SYBR	actA	Unkn-07	XylA SP2	20.96	20.91	0.228
B07	SYBR	aaglaA	Unkn-11	XylA SP2	15.54	15.39	0.197
C07	SYBR	aaglaA	Unkn-11	XylA SP2	15.16	15.39	0.197
D07	SYBR	aaglaA	Unkn-11	XylA SP2	15.46	15.39	0.197

Figure S6 – Quantification Cq results to determine the number of copies of the *aaglaA* gene

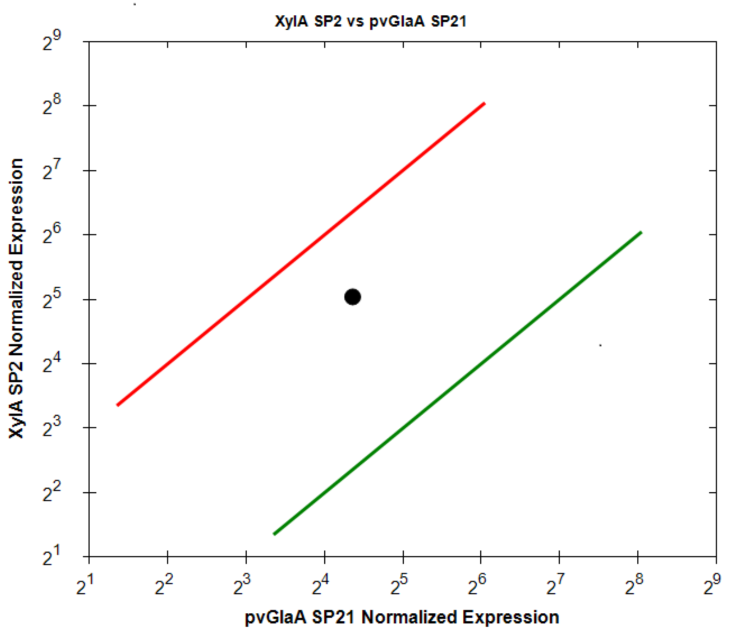

Displayed	Target	pvGlaA SP21 Normalized Expression	XylA SP2 Normalized Expression	XylA SP2 Fold Change	Target Regulation
Yes	aaglaA	20.53183	32.84878	1.59990	No change
Yes	actA				No change

Figure S7 - Gene expression results - scatter plot to determine the number of copies of the *aaglaA* gene

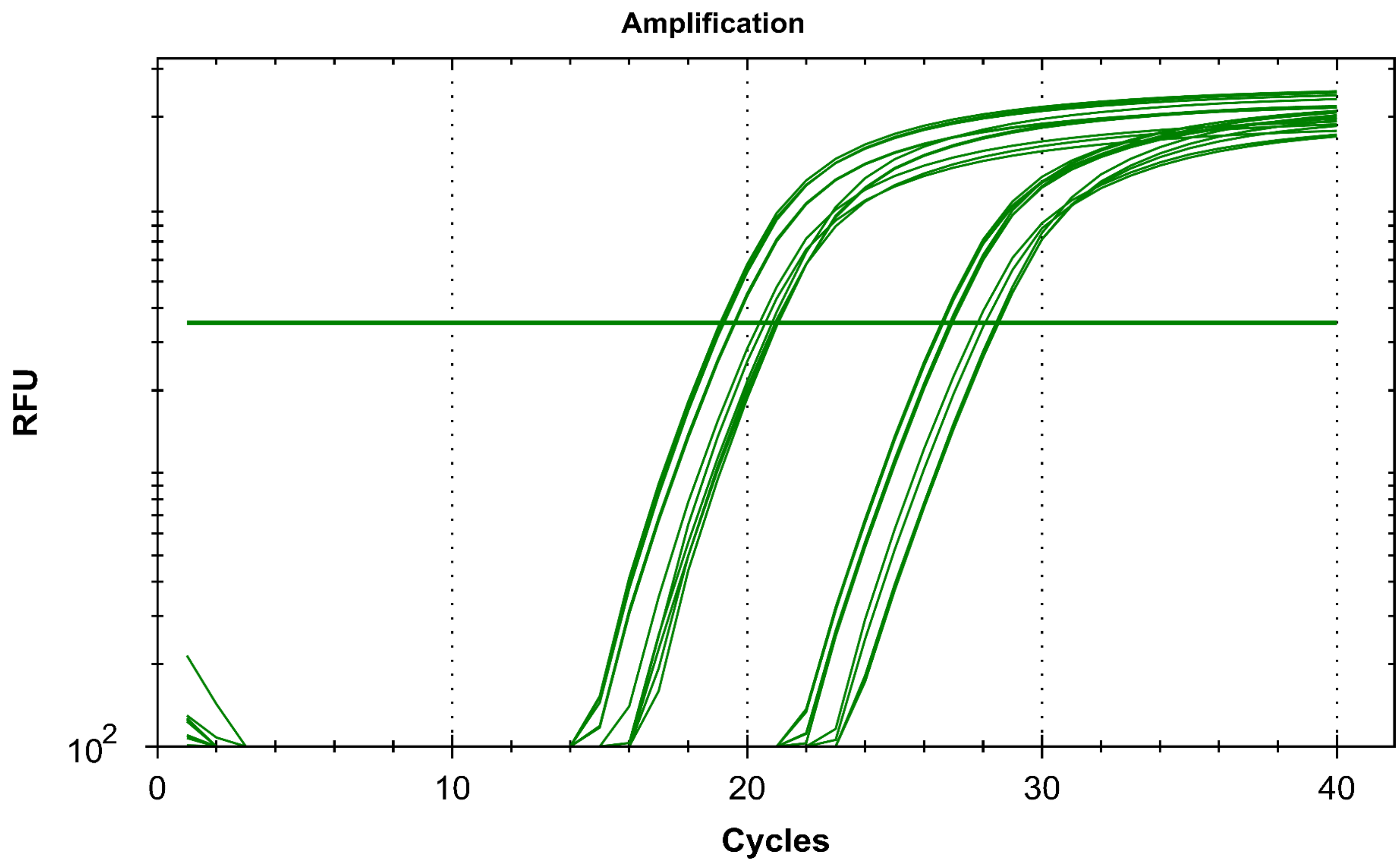

Amplification
RFU
10^2
0
10
20
30
40
Cycles

Well	Fluor	Target	Content	Sample	Cq	Cq Mean	Cq Std. Dev
A01	SYBR	actA	Unkn-01	aaGlaA SP6	26.55	26.55	0.032
A02	SYBR	actA	Unkn-01	aaGlaA SP6	26.58	26.55	0.032
A03	SYBR	actA	Unkn-01	aaGlaA SP6	26.52	26.55	0.032
A04	SYBR	actA	Unkn-02	Bgl1 SP2	28.05	27.92	0.196
A06	SYBR	actA	Unkn-02	Bgl1 SP2	27.78	27.92	0.196
A07	SYBR	actA	Unkn-03	pvGlaA SP21	26.84	26.89	0.053
A08	SYBR	actA	Unkn-03	pvGlaA SP21	26.87	26.89	0.053
A09	SYBR	actA	Unkn-03	pvGlaA SP21	26.94	26.89	0.053
A10	SYBR	actA	Unkn-04	XylA SP2	28.37	28.43	0.049
A11	SYBR	actA	Unkn-04	XylA SP2	28.46	28.43	0.049
A12	SYBR	actA	Unkn-04	XylA SP2	28.46	28.43	0.049
B01	SYBR	aaglaA	Unkn-05	aaGlaA SP6	19.13	19.12	0.053
B02	SYBR	aaglaA	Unkn-05	aaGlaA SP6	19.07	19.12	0.053
B03	SYBR	aaglaA	Unkn-05	aaGlaA SP6	19.17	19.12	0.053
B04	SYBR	aaglaA	Unkn-06	Bgl1 SP2	20.55	20.57	0.224
B05	SYBR	aaglaA	Unkn-06	Bgl1 SP2	20.80	20.57	0.224
B06	SYBR	aaglaA	Unkn-06	Bgl1 SP2	20.36	20.57	0.224
B07	SYBR	aaglaA	Unkn-07	pvGlaA SP21	19.49	19.51	0.020
B08	SYBR	aaglaA	Unkn-07	pvGlaA SP21	19.51	19.51	0.020
B09	SYBR	aaglaA	Unkn-07	pvGlaA SP21	19.53	19.51	0.020
B10	SYBR	aaglaA	Unkn-08	XylA SP2	20.94	21.00	0.053
B11	SYBR	aaglaA	Unkn-08	XylA SP2	21.01	21.00	0.053
B12	SYBR	aaglaA	Unkn-08	XylA SP2	21.04	21.00	0.053

Figure S8 – Quantification Cq results for the determination of *aaglaA* transcription level

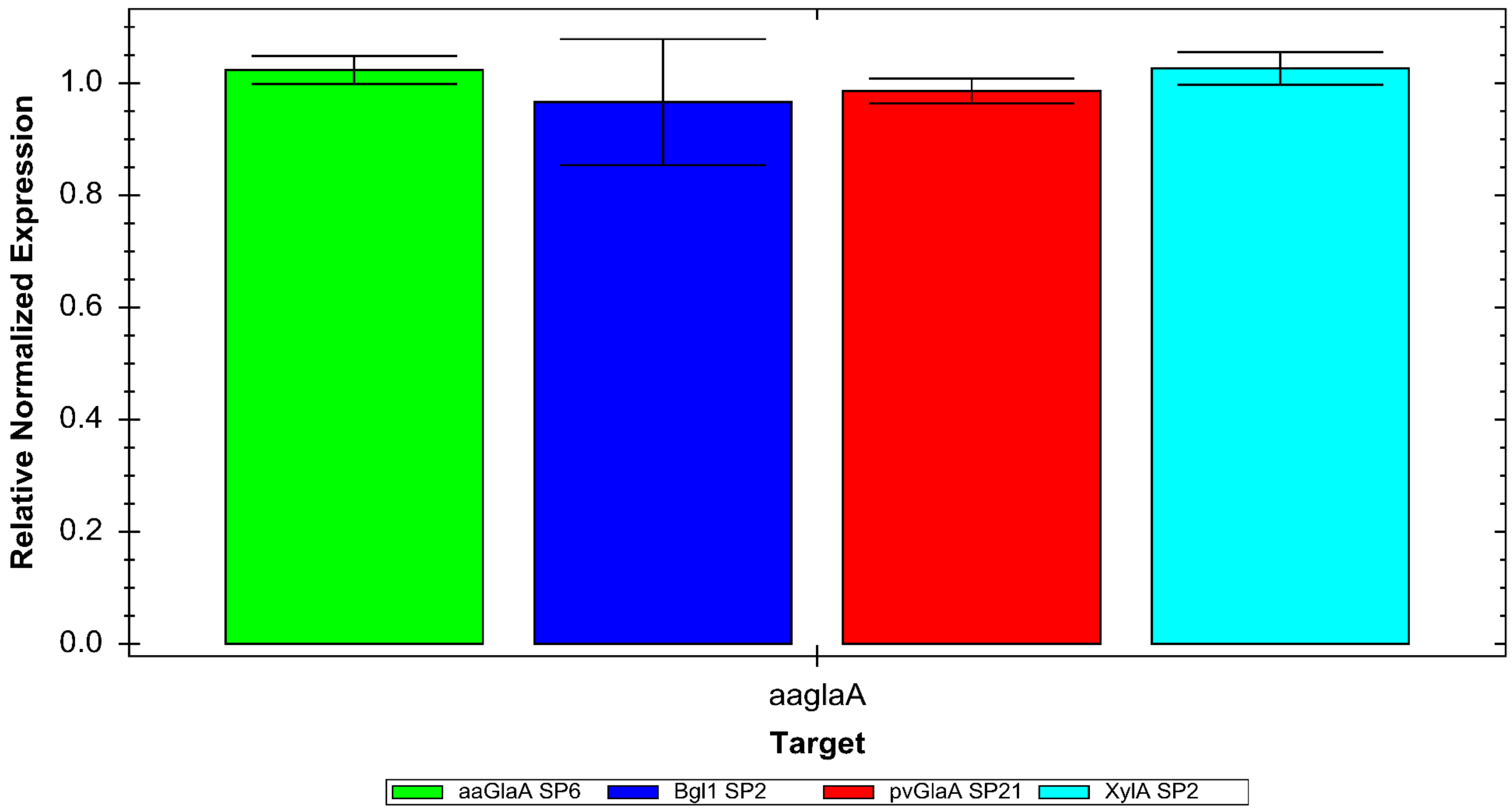

Target	Sample	Expression	Expression SEM	Corrected Expression SEM	Mean Cq	Cq SEM
aaglaA	aaGlaA SP6	1.02319	0.02477	0.02477	19.12	0.03072

aaglaA	Bgl1 SP2	0.96571	0.11244	0.11244	20.57	0.12922
aaglaA	pvGlaA SP21	0.98604	0.02204	0.02204	19.51	0.01167
aaglaA	XylA SP2	1.02637	0.02907	0.02907	21.00	0.03080
actA	aaGlaA SP6				26.55	0.01826
actA	Bgl1 SP2				27.92	0.13878
actA	pvGlaA SP21				26.89	0.03087
actA	XylA SP2				28.43	0.02828

Figure S9 – Gene Expression Results for aaglaA* gene in aaGlaA SP6 (green bar), Bgl1 SP2 (dark blue bar), pvGlaA SP21 (red bar) and XylA SP2 (light blue bar) strains.
